# *Enterococcus faecalis* Readily Adapts Membrane Phospholipid Composition to Environmental and Genetic Perturbation

**DOI:** 10.3389/fmicb.2021.616045

**Published:** 2021-05-21

**Authors:** Brittni M. Woodall, John R. Harp, William T. Brewer, Eric D. Tague, Shawn R. Campagna, Elizabeth M. Fozo

**Affiliations:** ^1^Department of Chemistry, University of Tennessee, Knoxville, Knoxville, TN, United States; ^2^Department of Microbiology, University of Tennessee, Knoxville, Knoxville, TN, United States; ^3^Biological and Small Molecule Mass Spectrometry Core, University of Tennessee, Knoxville, Knoxville, TN, United States

**Keywords:** lipidome, mprF2, lysyl-phosphatidylglycerol, cardiolipin, CLs, daptomycin

## Abstract

The bacterial lipid membrane, consisting both of fatty acid (acyl) tails and polar head groups, responds to changing conditions through alteration of either the acyl tails and/or head groups. This plasticity is critical for cell survival as it allows maintenance of both the protective nature of the membrane as well as functioning membrane protein complexes. Bacteria that live in fatty-acid rich environments, such as those found in the human host, can exploit host fatty acids to synthesize their own membranes, in turn, altering their physiology. *Enterococcus faecalis* is such an organism: it is a commensal of the mammalian intestine where it is exposed to fatty-acid rich bile, as well as a major cause of hospital infections during which it is exposed to fatty acid containing-serum. Within, we employed an untargeted approach to detect the most common phospholipid species of *E. faecalis* OG1RF via ultra-high performance liquid chromatography high-resolution mass spectrometry (UHPLC-HRMS). We examined not only how the composition responds upon exposure to host fatty acids but also how deletion of genes predicted to synthesize major polar head groups impact lipid composition. Regardless of genetic background and differing basal lipid composition, all strains were able to alter their lipid composition upon exposure to individual host fatty acids. Specific gene deletion strains, however, had altered survival to membrane damaging agents. Combined, the enterococcal lipidome is highly resilient in response to both genetic and environmental perturbation, likely contributing to stress survival.

## Introduction

The bacterial cellular envelope, consisting of the cell wall and membrane, is the first line of defense from environmental stresses (Silhavy et al., 2010). In particular, the cellular membrane that is composed primarily of lipids, is critical as it blocks the accumulation of toxins, senses changing conditions, houses protein signaling systems that are critical for stimulating gene expression changes, and provides a location for the major energetic complexes (Strahl and Errington, 2017). The majority of these lipids are phospholipids that consist of a polar head group and fatty acid (acyl) tails; though other lipid species, including sulfolipids and glycerophospholipids, exist and are vital for a variety of processes and functions across many species (Fozo and Rucks, 2016; Lopez-Lara and Geiger, 2017).

The lipid composition of any species is not static and bacteria will often alter their lipid composition in response to environmental perturbations. These alterations are needed to allow protein complexes to function properly. For example, as temperature decreases, so does molecular movement; bacteria will increase the proportion of monounsaturated fatty acids in their membranes to maintain fluidity under these conditions (de Mendoza, 2014; Fozo and Rucks, 2016; de Mendoza and Pilon, 2019). In regards to other stressors, a variety of fatty acid tail alterations can occur that are critical for survival. Additional studies have concluded that specific polar head groups are also necessary for stress survival (Fozo and Rucks, 2016). For example, depletion of cardiolipin in *Escherichia coli* leads to pleiotropic effects on its physiology (Rowlett et al., 2017).

*Enterococcus faecalis*, a Gram-positive bacterial commensal and pathogenic species, has been shown to alter its membrane fatty acid content in response to laboratory growth conditions (Saito et al., 2014, 2018; Harp et al., 2016; Brewer et al., 2020). Under these conditions, the fatty acid profile contains primarily the saturated fatty acid palmitic acid (C_16:0_) and an unsaturated fatty acid, *cis*-vaccenic acid (C_18:1_*_*cis*_*_11_). Within the host, *E. faecalis* is surrounded by the fatty acid rich fluids, bile (as an intestinal commensal) and serum (when pathogenic). Previous studies demonstrated that the organism will uptake the fatty acids from these fluids, in particular oleic acid (C_18:1_*_*cis*_*_9_) and linoleic acid (C_18:2_*_*cis*_*_9,12_), and utilize these species in its membrane (Saito et al., 2014; Harp et al., 2016). Supplementation with an individual fatty acid will lead to the provided species dominating the fatty acid composition, which can have significant impacts on cellular growth, morphology, and stress responses (Saito et al., 2018; Brewer et al., 2020). This supplementation also leads to a decrease in native enterococcal fatty acids found within the membrane.

The above-mentioned studies have focused primarily on the fatty acid tail alterations that can occur, and fewer studies have focused on understanding the changes in intact lipid species. In *E. faecalis*, along with presence of free fatty acids within the membrane, several lipid head groups have been confirmed: phosphatidylglycerol (PG), lysyl-phosphatidylglycerol (L-PG), cardiolipin (CL), diacylglycerol (DAG), and monoglucosyl-diacylglycerol (MGDG) (Ibbott and Abrams, 1964; Mishra et al., 2012; Hines et al., 2017; Rashid et al., 2017; Tague et al., 2019). The DAGs and MGDGs (a group of glycolipids) are generated during lipoteichoic acid formation in Gram positive bacteria (Zhang and Rock, 2008; Van Horn and Sanders, 2012). Of the phospholipid species, phosphatidylglycerol is dominant in the membrane and serves as a precursor for both CL and L-PG. Cardiolipin is typically derived from the addition of two phosphatidylglycerol moieties to a bridging glycerol, leading to a phospholipid that contains four fatty acid tails (Schlame, 2008). L-PG is also derived from PG and is formed from the addition of a lysine to the headgroup (Peschel et al., 2001; Roy and Ibba, 2008). The proportion of these specific lipid classes can change during growth in liquid culture. For example, specific PG species decreased as the culture entered stationary phase, while L-PG increased (Rashid et al., 2017). A study by Tague et al. (2019) also demonstrated that prolonged exposure to oleic or linoleic acid led to changes in the proportion of PG and L-PG species, as well as accumulation of either fatty acid within the membrane of *E. faecalis* OG1RF. Others have shown that an enterococcal strain resistant to the antibiotic daptomycin had reduced levels of PG species compared to its sensitive derivative, suggesting that genetic perturbations will also alter lipid content (Mishra et al., 2012; Hines et al., 2017).

Given these findings, the plasticity of the phospholipid composition of *E. faecalis* was examined using untargeted lipidomics to monitor membrane changes in response to environmental and genetic perturbations. An ultra-high performance liquid chromatography high-resolution mass spectrometry (UHPLC-HRMS) approach was used to identify those tail and polar head group combinations most likely to occur within the membrane: this allowed us to detect as many as 63 species (5 free fatty acids, 9 PGs, 4 L-PGs, 11 CLs, 12 DAGs, 16 MGDGs). Specifically, the response of the organism to changing environments through short-exposures to host-derived fatty acids was examined and strains deleted for genes in phospholipid biosynthesis were used to better determine how the organism would compensate for the loss of specific lipid synthetic capabilities.

## Materials and Methods

### Bacterial Growth Conditions

*Enterococcus faecalis* strains were grown statically in brain heart infusion medium (BHI; BD Difco) at 37°C. For the determination of generation time (long-term supplementation), overnight cultures were diluted into fresh BHI medium to an optical density at 600 nm (OD_600_) of 0.01 with either oleic acid (C_18:1_*_*cis*_*_9_, 20 μg ml^–1^), linoleic acid (C_18:2_*_*cis*_*_9,12_, 10 g ml^–1^) or solvent control (equal volume ethanol) and grown until stationary phase (Saito et al., 2014). For all other fatty acid exposure experiments, referred to as short-term supplementation and used for lipid compositional analyses, overnight cultures were diluted into fresh BHI medium as described above and grown until an OD_600_ of ∼0.25 (Saito et al., 2018). Fatty acid supplements [20 μg ml^–1^ oleic acid (C_18:1_*_*cis*_*_9_) or 10 μg ml^–1^ linoleic acid (C_18:2_*_*cis*_*_9,12_)] or an equal volume of ethanol was added and cultures were incubated at 37°C for an additional 30 min to eliminate any possible negative effects on growth (Harp et al., 2016). All fatty acids and chemicals were purchased from Millipore-Sigma unless noted otherwise. Counter-selection for *E. faecalis* cultures was performed on MM9YEG agar plates (final concentration: 1X M9 salts, 0.25% yeast extract, 250 μg ml^–1^ 5-bromo-4-chloro-3-indolyl-β-D-galactopyranoside (X-gal), and 0.5% glucose) containing 10 mM *p-*Cl-phenylalanine (*p*-Cl-Phe). *Escherichia coli* strains were grown in LB medium at 37°C with shaking. Antibiotics were used at the following concentrations when needed: erythromycin, 10 μg ml^–1^ (*E. faecalis*) or 100 μg ml^–1^ (*E. coli*); spectinomycin, 1,000 μg ml^–1^; fusidic acid, 25 μg ml^–1^; rifampicin, 250 μg ml^–1^.

### Generation of Bacterial Deletion Strains

The strains and plasmids used in this study are listed in Supplementary Table 1 and the sequence of all oligonucleotides are provided in Supplementary Table 2. Generation of deletion strains of *E. faecalis* OG1RF was through the method of Kristich et al. (2007). To delete the *mprF2* gene and two predicted cardiolipin synthase genes, ∼1,000 bp flanking either OG1RF_RS03930 (*mprF2*), OG1RF_RS01975 (*cls1*) or OG1RF_RS06840 (*cls2*) were amplified from *E. faecalis* OG1RF genomic DNA. The two products for each gene region were then spliced together using the external primers. Primers containing complementary overlaps with the spliced gene regions were used to amplify pCJK47 (Kristich et al., 2007). The amplified inserts and vectors were assembled using NEB Gibson Assembly Master Mix. The assembled products were transformed into *E. coli* strain EC1000. Once verified, pMprF2 (to generate Δ*mprF2*), pJRH1 (to generate Δ*cls1*) or pJRH2 (to generate Δ*cls2*) was transformed into an *E. faecalis* conjugative donor strain (CK111/pCF10-101). After clonal selection, conjugative donors containing pMPRF2, pJRH1 or pJRH2 were mixed with an OG1RF recipient at a ratio of 1-part donor to 9 parts recipient. After conjugation, cells were placed on recipient (rifampicin, fusidic acid, erythromycin and X-gal) or donor (spectinomycin and erythromycin) selection media. Blue colonies from the recipient plates were then re-isolated on the same selective media. Isolated colonies were grown to stationary phase in BHI in the absence of selection, diluted, and then isolated on MM9YEG. White colonies were tested for erythromycin sensitivity and sequenced for verification.

### GC FAME Preparation and Analysis

Cells were grown via short term supplementation as outlined in bacterial growth conditions above. Fifteen milliliter aliquots of cells were harvested via centrifugation (2739×*g* for 10 min), washed twice with 10 ml of 1X phosphate buffered saline (PBS), pelleted, and stored at −80°C prior to shipment to Microbial ID, Inc. (Newark, DE). Cells were then subjected to saponification with a sodium hydroxide-methanol mixture, a methylation step, and hexane extraction prior to GC-FAME analysis (Sasser et al., 2005).

### Membrane Challenge Assays

Cells were diluted to an OD_600_ of 0.01 in BHI medium, incubated until exponential phase (OD_600_ of ∼0.225 to 0.25), and then supplemented with either solvent control, 20 μg ml^–1^ oleic acid (C_18:1_*_*cis*_*_9_) or 10 μg ml^–1^ linoleic acid (C_18:2_*_*cis*_*_9,12_) for 30 min (Saito et al., 2018). Ten milliliter of cells were harvested and washed twice with 10 ml of 1X PBS and then resuspended in BHI (SDS challenge) or BHI containing 1.5 mM CaCl_2_ (daptomycin challenge) and treated with either a final concentration of 0.05% SDS or 15 μg ml^–1^ of daptomycin, given the inherent sensitivity of the Δ*cls1/cls2* strain. Serial dilutions were plated onto BHI agar at the times indicated in the text. The log ratio of survivors over time was calculated for *n* = 3 biological replicates and shown are the averages and standard deviations for each experiment.

### Phospholipid Extraction for UHPLC-HRMS Analysis

Cells were grown as indicated above and 5 ml were harvested by centrifugation (2739×*g* for 10 min). The supernatants were removed and the remaining pellets were washed with 15 ml of PBS and stored in conical vials at −80°C prior to lipid extraction. Lipids were extracted using a method of Guan et al. (2010) with modifications. Briefly, pellets were brought to room temperature and resuspended in 5 ml of extraction solvent (95% ethanol, water, diethyl ether, pyridine, 4.2 N ammonium hydroxide; 15:15:5:1:0.18). Glass beads were added and cells were vortexed for 30 s before being heated at 60°C for 1 h. After incubation, the organic solvent was removed via pipetting and placed in a respectively, labeled glass dram vial, leaving glass beads behind. An additional 5 ml of extraction buffer was added to the vial, contents were vortexed and incubated for another hour at 60°C. At this time, the organic extracts were combined and dried under a stream N_2_. The resulting solids were resuspended in 300 μL of 9:1 methanol:chloroform and stored at −20°C until UHPLC-HRMS analysis.

### Ultra-High Performance Liquid Chromatography High Resolution Mass Spectrometry (UHPLC-HRMS)

Lipid profiling was performed in the manner of Bird et al. (2011) with modification. Samples were stored at 4°C prior to analysis. An UltiMate 3000 ultra-high performance liquid chromatography system (UHPLC, Dionex, Sunnyvale, CA) was used to inject 10 μl of sample onto a CORTECS C18 column (90 Å, 2.7 μm, 2.1 mm × 150 mm; Waters) controlled at 40°C. Mobile phase A was 60:40 acetonitrile/water with 10 mM ammonium formate as a buffer and 0.1% formic acid while mobile phase B consisted of 90:10 2-propanol/acetonitrile with 10 mM ammonium formate as a buffer and 0.1% formic acid. The gradient used follows: *t* = 0 min: 32% solvent B flow rate of 0.30 ml/min, *t* = 1.5 min: 32% solvent B flow rate 0.4 ml/min, *t* = 2.5 min: 45% solvent B flow rate 0.4 ml/min, *t* = 5 min: 52% solvent B flow rate 0.3 ml/min, *t* = 8.0 min 58% solvent B flow rate 0.3 ml/min, *t* = 11.0 min 68% solvent B flow rate 0.4 ml/min, *t* = 14.0 min 75% mobile phase B flow rate of 0.4 ml/min, *t* = 18.0 min 80% solvent B flow rate of 0.4 mL/min, *t* = 21 98% solvent B flow rate 0.45 ml/min, *t* = 25.0 min 32% solvent B flow rate 0.3 ml/min until equilibration at 30 min. Eluent was introduced to the mass spectrometer via an electrospray ionization (ESI) source, with the following parameters: sheath gas 30 (arbitrary units), aux gas 8 (arbitrary units), sweep gas 3 (arbitrary units), spray voltage 3 kV, capillary temperature 300°C. Mass analysis was performed using an Exactive Plus (Thermo Scientific, Waltham, MA) mass spectrometer operated in dual polarity mode. Ions of respective DAG and MGDG species were detected in positive mode while free fatty acids, PG, L-PG, and CL were detected in negative mode. Masses were detected in full scan mode within a scan range of 100–1,500 *m/z*, operated at a resolution of 140,000, with an automatic gain control target of (AGC) of 3 × 10^6^ ions, and a maximum IT time of 100 ms. Full scan data was complemented with all ion fragmentation data at a resolution of 35,000 utilizing 35 eV collisional energy. The mass spectrometer was calibrated every 24 h.

### Data Processing and Statistical Analysis

A list of potential lipids was created using GC FAME data to determine which fatty acids were expected within the biological samples (Supplementary Table 3; Ibbott and Abrams, 1964; Saito et al., 2014, 2018). This list of chemical formulas allowed detection of 6 lipids classes based on calculated mass. External standards were analyzed to verify retention time and charge state. External standards included MGDG (34:1), DAG (36:2), DAG (36:4), PG (36:0), L-PG (36:2), and CL (72:4). All lipid standards were purchased from Avanti Polar Lipids Inc. (Alabaster, AL). Raw data acquired from Xcalibur were converted to mzML format utilizing MS convert from ProteoWizard. El MAVEN was utilized to integrate exact masses of predicted lipid species with an error of less than 5 ppm. The integrated intensities were normalized to the optical density (OD_600_ nm) of the culture at the time of extraction. A Grubbs’ test was utilized to exclude any outliers within the biological replicates in the data set. An alpha value of 1.67 was chosen for the Grubbs test to represent a confidence interval of 95%. After Grubbs analysis, outliers residing outside the 95% confidence interval were removed; the average peak area, standard deviation, and coefficient of variation were then calculated. Any remaining data points with a representative coefficient variation greater than 30% were removed to aid in confirmation of presented data. Heatmaps were constructed by determining the ratio of normalized, log transformed intensities of experimental values over control values. A paired student’s *t*-test was utilized to determine significance. Three dots denote *P* < 0.01; two dots indicate *P*-value between 0.01 and 0.05; one dot, *P*-value between 0.05 and 0.1. In order to ensure the *p*-values were not contributing to a high false discovery rates, data was inputted into Metaboanalyst (mataboabalyst.ca) and analyzed via one-way ANOVA and *post hoc* tests. Percentage of total graphs were constructed by summing ion totals of all detected lipids into specific lipid categories and presenting respective lipid classes as a percentage of total ions detected.

## Results

### Supplementation With Host Fatty Acids Increases Specific Lipid Species According to Tail Group

Previous research utilizing fatty acid methyl ester (FAME) analysis has shown that *E. faecalis* OG1RF can incorporate exogenous fatty acids including oleic acid and linoleic acid from host fluids, altering its membrane fatty acid content and sensitivity to membrane damaging agents (Saito et al., 2014, 2018; Harp et al., 2016; Brewer et al., 2020). However, alterations to intact lipid species consisting of head group and fatty acid tails were not examined. To address this question, we examined lipid species of OG1RF using UHPLC-HRMS (Materials and Methods) and GC-FAME analysis (Supplementary Table 5). Cells were subject to brief exposure to one of three conditions: solvent control (ethanol), oleic acid, or linoleic acid, as these host-derived fatty acids are readily utilized by the organism and trigger stress protection (Saito et al., 2018; Brewer et al., 2020). Short-exposures were also used as the addition of linoleic acid in particular, impacted generation time if given at the time of dilution (see below; Saito et al., 2018). The lipid species detected in this analysis are listed with their chemical formulas and can be found in Supplementary Table 3. While LC-MS can detect thousands of unique masses, we focused specifically on these species as they represent the most commonly detected tail species and headgroups (Ibbott and Abrams, 1964; Saito et al., 2014, 2018; Hines et al., 2017; Rashid et al., 2017; Tague et al., 2019).

**FIGURE 1:**
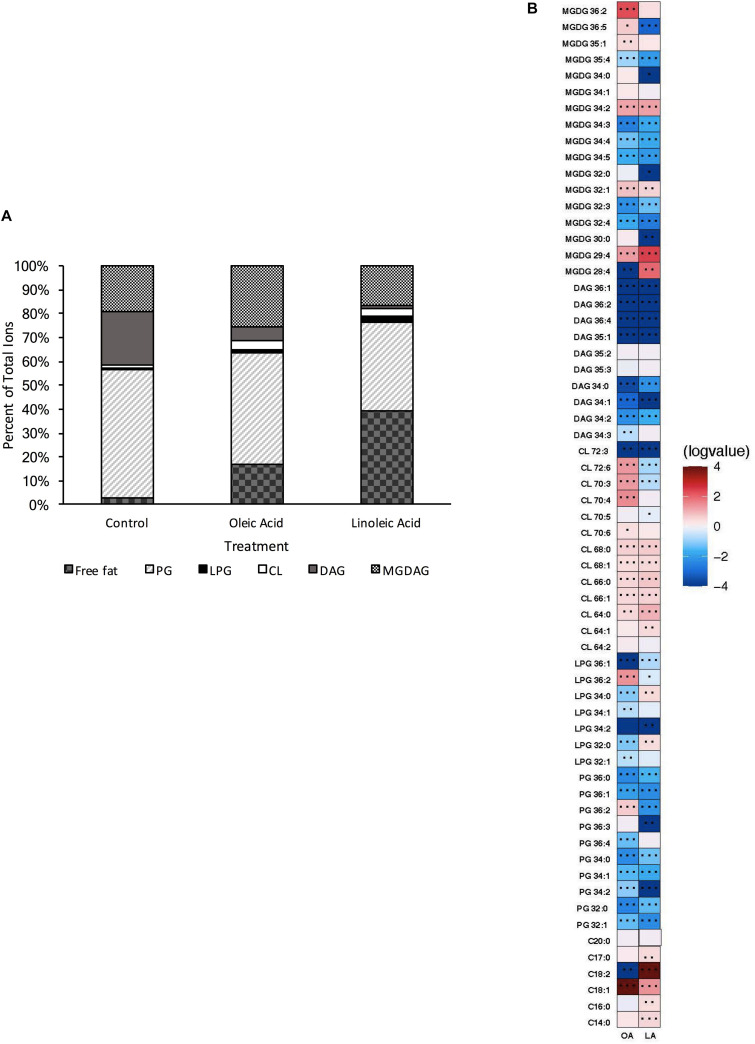
*Enterococcus faecalis* OG1RF lipid composition is altered upon exposure to host fatty acids. **(A)** Percent totals of lipid species in solvent control: free fatty acids (2.7%), PG (53.7%), L-PG (1.1%), CL (1.1%), DAG (22.4%), and MGDG (19.0%), oleic acid: free fatty acids (16.8%), PG (46.7%), L-PG (1.5%), CL 3.3%), DAG (6.1%), and MGDG (25.6%), linoleic acid: free fatty acids (39.5%), PG (36.6%), L-PG (2.6%), CL (3.4%), DAG (1.4%), and MGDG (16.6%). Species were normalized to OD_600 *nm*_. **(B)** Heat-map of fold changes upon fatty acid supplementation vs. solvent control; OA, oleic acid supplementation; LA, linoleic acid supplementation. Shown are the average fold changes of those detected lipid species above limit of detection for *n* = 5 samples. *P = 0.1–0.05; **P = 0.05–0.01; ***P < 0.01.

Under our control conditions, OG1RF had a lipid profile dominated by PGs (54% of total species), although DAGs (22% of total), MGDAGs (19% of total), free fatty acids (3% of total), L-PGs (1% of total), and CLs (1% of total) were also detected (Figure 1A). When examining which individual species were most prevalent, we noted PG 32:0, PG 34:1, PG 34:2 and PG 36:2 were detected at high levels (integrated area > 10^7^): these species corresponded well with the GC-FAME analyses of fatty acid tail profiles with C_16:0_ and C_18:1_*_*cis*_*_11_ being the dominant tails (Supplementary Table 4). Similarly, CL 66:1, DAG 34:1, DAG 36:2, MGDG 34:1 were all highly expressed as well.

While PG species are responsible for nearly 54% of the lipids detected in this method for our growth conditions (Supplementary Table 3), upon short term supplementation with oleic (C_18:1_*_*cis*_*_9_) or linoleic (C_18:2*cis*9,12_) acids, found in host fluids bile and serum, there were major alterations in the contribution of the individual lipid classes compared to the control (Figures 1A,B). Cells pulsed with oleic acid were increased in free fatty acids, CL, and MGDG, with a resulting decrease in PG and DAG species, based on total ion counts. There was a large increase in the amount of free C_18:1_ detected in the membrane, which correlates with the GC-FAME data where the combined C_18:1_ species (consisting of both native *cis*-vaccenic acid, C_18:1_*_*cis*_*_11_ and oleic acid, C_18:1_*_*cis*_*_9_) comprised over 65% of total fatty acids detected after oleic acid supplementation, while these combined species were only 40% in the control cultures (Supplementary Table 4). Lipid species correlating to one or more C_18:1_ tails were also increased including, PG 36:2, L-PG 36:2, CL 70:4, CL 70:3, and MGDG 36:2 (Figure 1B; please refer to the legend for *P*-values). Other species that likely did not possess oleic acid tails were significantly increased including CL 68:1, CL 68:0, CL 72:6, MGDG 35:1, MGDG 34:2, MGDG 32:1, and MGDG 26:4.

Short term exposure to linoleic acid also resulted in large alterations of the lipid profile from control. This included large increases in the proportion of detected free fatty acids and cardiolipin species; like supplementation with oleic acid, there was an overall decrease in PG species compared to control (Figure 1A). The amount of free C_18:2_ within the membrane was a major driver for the detected free fatty acids within the membrane (Figure 1B; integrated area > 10^8^), again corresponding to the GC-FAME analysis (Supplementary Table 4; 24% of total fatty acids detected via this method). While there was an increase in specific cardiolipin species upon linoleic acid supplementation, these species (CL 64:0, CL 64:1, CL 66:0, CL 66:1, CL 68:0, CL 68:1; Figure 1B) would not possess a linoleic acid tail; however, many were also conserved upon oleic acid supplementation.

Given the increases in cardiolipin species upon supplementation with either oleic or linoleic acid both fatty acids, we hypothesized these changes may be critical for maintaining proper membrane function in these growth environments and thus may contribute to stress responses (Arias et al., 2011; Koprivnjak et al., 2011; Palmer et al., 2011; Mishra et al., 2012; Davlieva et al., 2013). Therefore, we generated a strain deleted for the two genes predicted to be responsible for cardiolipin synthesis: cardiolipin synthase 1 (ΔOG1RF_RS01975—Δ*cls1*) and cardiolipin synthase 2 (ΔOG1RF_RS06840—Δ*cls2*).

### Deletion of Both Cardiolipin Synthase Genes Is Required for Full Depletion of Cardiolipin Species

**FIGURE 2:**
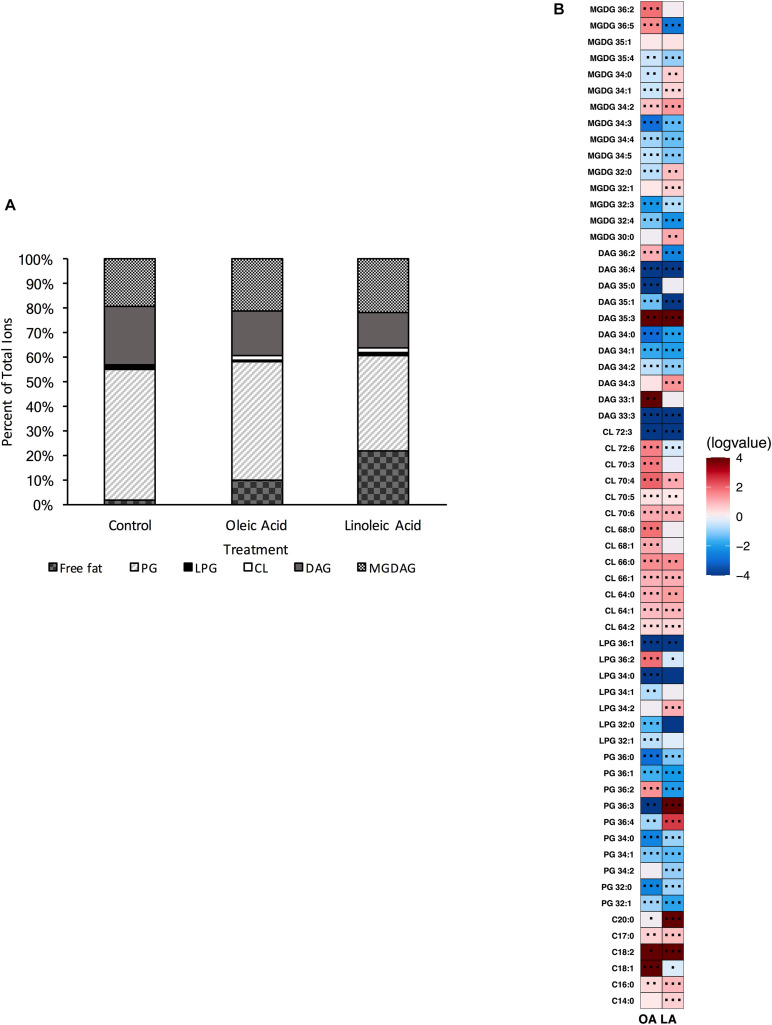
OG1RF lacking Δ*cls1* can still produce cardiolipin and alter its lipid composition upon exposure to host fatty acids. **(A)** Percent totals of lipid species for Δ*cls1* in solvent control: free fatty acids (2.0%), PG (53.5%), L-PG (0.7%), CL (0.7%), DAG (23.8%), and MGDG (19.4%), oleic acid: free fatty acids (9.8%), PG (48.3%), L-PG (0.7%), CL 2.0%), DAG (17.9%), and MGDG (21.3%), linoleic acid: free fatty acids (22.1%), PG (38.9%), L-PG (1.2%), CL (1.8%), DAG (14.7%), and MGDG (21.4%). Species were normalized to OD_600 *nm*_. **(B)** Heat-map of fold changes upon fatty acid supplementation vs. solvent control for Δ*cls1*; OA, oleic acid supplementation; LA, linoleic acid supplementation. Shown are the average fold changes of those detected lipid species above limit of detection for *n* = 5 samples. *P = 0.1–0.05; **P = 0.05–0.01; ***P < 0.01.

**FIGURE 3:**
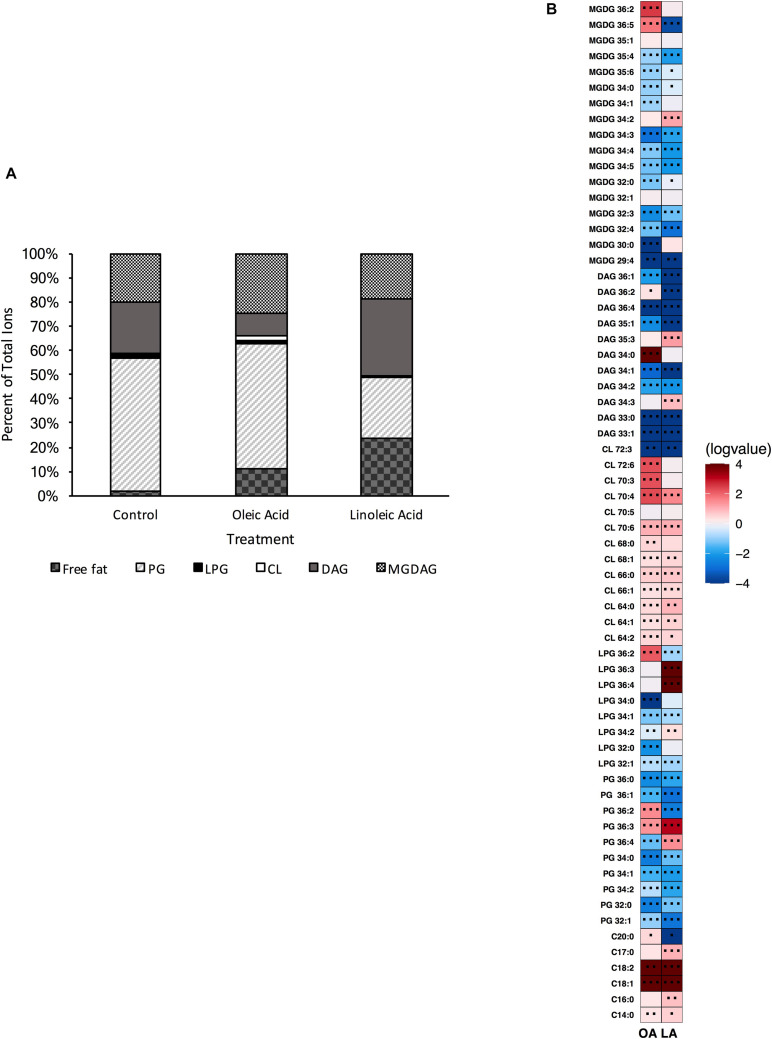
OG1RF lacking Δ*cls2* can still produce cardiolipin and alter its lipid composition upon exposure to host fatty acids. **(A)** Percent totals of lipid species for Δ*cls2* in solvent control: free fatty acids (1.9%), PG (55.1%), L-PG (1.0%), CL (0.7%), DAG (21.2%), and MGDG (20.1%), oleic acid: free fatty acids (11.1%), PG (51.6%), L-PG (1.2%), CL 2.1%), DAG (9.4%), and MGDG (24.6%), linoleic acid: free fatty acids (35.5%), PG (35.5%), L-PG (1.9%), CL (2.6%), DAG (4.1%), and MGDG (20.4%). Species were normalized to OD_600 *nm*_. **(B)** Heat-map of fold changes Δ*cls2* upon fatty acid supplementation vs. solvent control; OA, oleic acid supplementation; LA, linoleic acid supplementation. Shown are the average fold changes of those detected lipid species above limit of detection for *n* = 5 samples. *P = 0.1–0.05; **P = 0.05–0.01; ***P < 0.01.

Both single gene deletion strains, Δ*cls1* and Δ*cls2* were analyzed via UHPLC-HRMS. Each strain was able to produce detectable levels of cardiolipin; however, when compared to the parental OG1RF strain, we noted a significant reduction in all CL (Supplementary Figure 1). However, the deletion strains did not necessarily have other changes in common (Figures 2A, 3A). For Δ*cls1*, we noted significantly higher levels of free C18:1 while Δ*cls2* had increased PG 32:0, PG 36:3 and several MGDG species (Figures 2B, 3B).

Supplementation with either oleic or linoleic acid led to an accumulation of many CL species in both single gene deletion strains. Similarly, the deletion strains, like the parental strain, had elevated levels of PG 36:2 and L-PG 36:2, as well as free C_18:1_ within their membranes when provided oleic acid (Figures 1B, 2B, 3B), which corresponded with the increase in total C_18:1_ measured via GC-FAME (Supplementary Table 4). Linoleic acid, however, resulted in the accumulation of specific species, dependent upon the genetic background of the strain. While both Δ*cls1* and Δ*cls2* accumulated PG 36:3 and PG 36:4, Δ*cls2* also accumulated L-PG 36:3 and L-PG 36:4: note that none of these four species were increased in the parental OG1RF strain when given linoleic acid (Figures 1B, 2B, 3B). Likely these species contained C_18:2_ tails and both strains accumulated C_18:2_ as determined via GC-FAME analysis (Supplementary Table 4) and our UHPLC-HRMS method. So while there were some unique alterations in the lipid content upon deletion of *cls1* vs. *cls2*, both strains could clearly produce cardiolipin. Therefore, we generated the double deletion strain, Δ*cls1/cls2* to better examine the effects of cardiolipin loss on the lipidome as well as enterococcal physiology.

**FIGURE 4:**
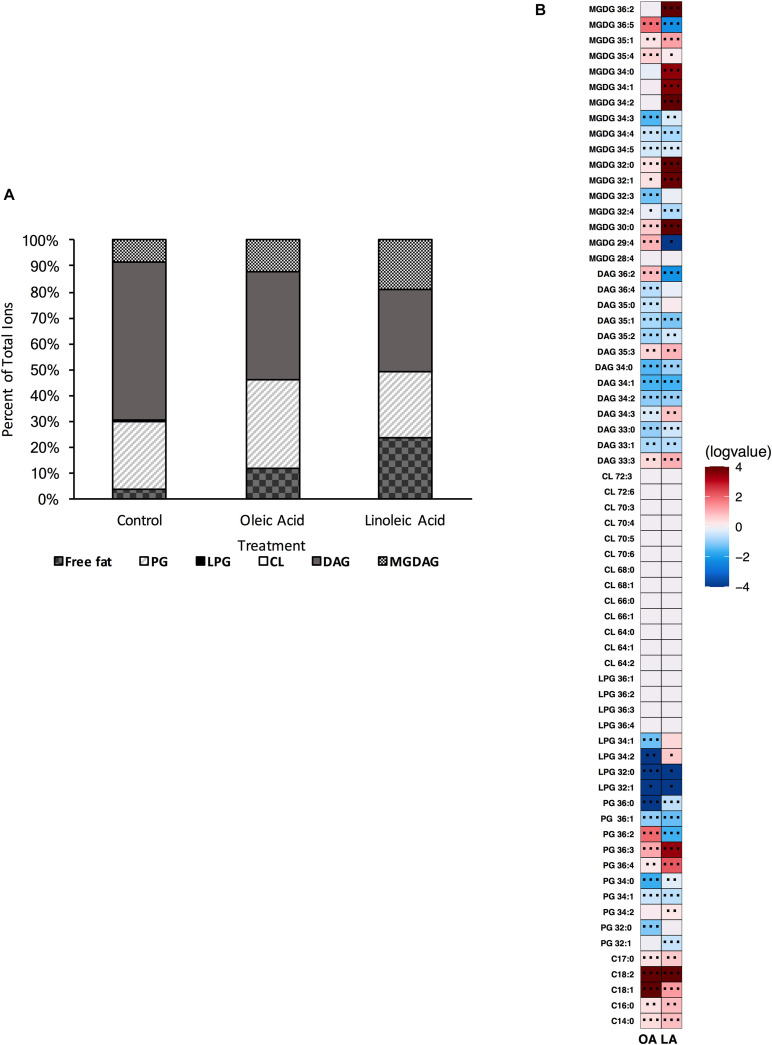
A strain deleted for Δ*cls1/cls2* can alter its lipid composition upon exposure to host fatty acids. **(A)** Percent totals of lipid species in solvent control: free fatty acids (3.8%), PG (26.3%), L-PG (0.1%), CL (0.0%), DAG (61.3%), and MGDG (8.5%), oleic acid: free fatty acids (11.7%), PG (34.3%), L-PG (0.0%), CL 0.0%), DAG (41.5%), and MGDG (12.5%), linoleic acid: free fatty acids (23.4%), PG (25.7%), L-PG (0.2%), CL (0.0%), DAG (31.9%), and MGDG (19.0%). Species were normalized to OD_600 *nm*_. **(B)** Heat-map of fold changes upon fatty acid supplementation vs. solvent control; OA, oleic acid supplementation; LA, linoleic acid supplementation. Shown are the average fold changes of those detected lipid species above limit of detection for *n* = 5 samples. *P = 0.1–0.05; **P = 0.05–0.01; ***P < 0.01.

The resultant strain was unable to produce cardiolipin, as confirmed via UHPLC-HRMS (Supplementary Figure 2); thus both genes are bona fide *cls* synthases and contribute to its production. In examining the basal membrane profile, we saw enrichment of most targeted PG and DAG species when compared to the parental strain, and several MGDG species (Supplementary Figure 3). Surprisingly, not only was the strain lacking cardiolipin, we noted significant reduction in most L-PG species as well, suggesting that some of the PG accumulation was in part due to a lack of L-PG synthase activity (Supplementary Figure 3). We note that there were major shifts in the membrane composition of Δ*cls1/cls2* compared to the parental wildtype strain (Figure 4A vs. Figure 1A), yet CL comprised only a minor portion of the species detected under basal conditions (Figure 1A).

Supplementation of Δ*cls1/cls2* with oleic acid resulted in increased amounts of free fatty acids (C_14:0_, C_16:0_, C_18:1_, C1_8:2_, and C_17:0_), PG species (in particular PG 36:2, PG 36:3), with the higher amounts of C_18:1_ tails corresponding to the abundance of oleic acid and *cis*-vaccenic acid detected via GC-FAME (Supplementary Table 4). Several MGDG species (MGDG 29:4, MGDG 30:0, MGDG 32:0, MGDG 35:4, MGDG 35:1, and MGDG 36:5) were also elevated when compared to solvent control. For these lipid species, only PG 36:2, MGDG 29:4, MGDG 35:1 and MGDG 36:5 were also induced in a wild type background. A few minor increases in specific DAGs were also increased (Figure 4B) with oleic acid supplementation, however, the overall percent of DAG in the membrane decreased from 61 to 42% (Figure 4A).

The membrane profile of Δ*cls1/cls2* when provided linoleic acid was shifted from solvent control: linoleic acid exposed cells had far more free fatty acids and MGDAGs comprising the membrane with a large reduction in DAG species (Figure 4A). Linoleic acid supplementation decreased total PG species compared to ethanol, but showed much higher increases in free fatty acids and MGDG species compared to solvent control. When examining individual species, there was a large increase in C_18:2_ within the membrane, in agreement with the GC-FAME analysis (Figure 4B and Supplementary Table 4). Several MGDG species were increased with linoleic acid, as was seen with oleic acid including, MGDG 30:0, MGDG 32:0, MGDG 32:1, MGDG 35:4 and MGDG 35:1 (Figure 4B). While the proportion of the membrane composed of PG species remained relatively constant, there was significant accumulation of both PG 36:3 and PG 36:4 which could include linoleic acid tails.

Given that we were able to alter the membrane composition of *E. faecalis* with both genetic and environmental perturbations, we were interested probing the sensitivity of this altered membrane in response to membrane disrupting agents.

### The Loss of Both Cardiolipin Synthase 1 and 2 Impacts Sensitivity to Membrane Damaging Agents

**FIGURE 5:**
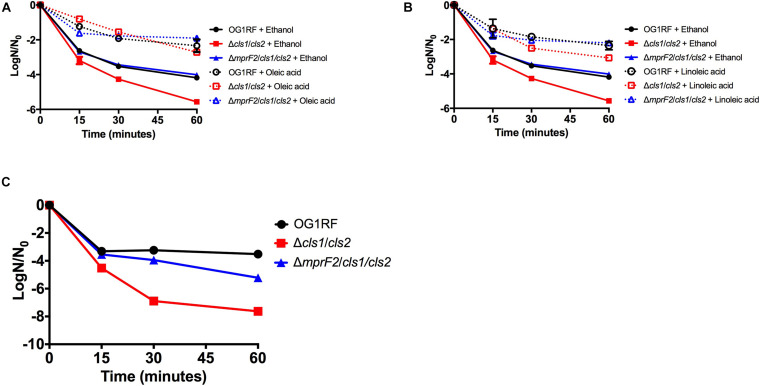
Stress sensitivity upon loss of phospholipid synthetic pathways is dependent upon genetic background. **(A)** Oleic acid supplementation of Δ*cls1/cls2* and Δ*mprF2/cls1/cls2* had statistically increased numbers of survivors vs. the solvent control when treated with daptomycin at all-time points (*P* < 0.0001). **(B)** Linoleic acid supplementation of Δ*cls1/cls2* and Δ*mprF2/cls1/cls2* had statistically increased numbers of survivors vs. the solvent control when treated with daptomycin at all-time points (*P* = 0.0001). Shown are the averages ± standard deviation for *n* = 3. **(C)** SDS sensitivity of indicated strains grown in exponential phase. Shown are the averages ± standard deviation for *n* = 3.

Alterations in cardiolipin synthase activity or in the levels of cardiolipin have been implicated in sensitivity to the antibiotic daptomycin: specifically, increased CL levels potentially reduce the effectiveness of the drug as it does not allow for proper drug interaction with the membrane (Arias et al., 2011; Palmer et al., 2011; Mishra et al., 2012; Davlieva et al., 2013; Zhang et al., 2014). Given our Δ*cls1/cls2* strain lacked cardiolipin, we first examined its sensitivity to daptomycin. The Δ*cls1/cls2* strain had increased basal sensitivity to daptomycin (*P* = 0.002) compared to parental OG1RF (Figures 5A,B). This sensitivity was dependent upon deletion of both genes, as single gene deletion strains did not impair survival (Supplementary Figure 4).

We previously demonstrated that the addition of either oleic or linoleic acid could induce a physiological tolerance to daptomycin in OG1RF: cells exposed to either fatty acid survived high concentrations of the drug far better than those without (Saito et al., 2018; Brewer et al., 2020). Given that oleic or linoleic acid also result in increases in cardiolipin in the parental strain, and Δ*cls1/cls2* lacks cardiolipin, we hypothesized that oleic or linoleic acid addition would not protect our cardiolipin null strain from daptomycin. However, addition of either fatty acid did induce tolerance in Δ*cls1/cls2*, yet the protection was not as long lasting as in the parental OG1RF strain (Figures 5A,B and Supplementary Figure 5). To conclude whether Δ*cls1/cls2* was simply more inherently sensitive to membrane damage, we examined its tolerance to the detergent SDS. The double deletion strain was far more sensitive to SDS than the parental strain (Figure 5C). We also noted increased generation time of Δ*cls1/cls2* during long-term growth in linoleic acid (see Materials and Methods and Supplementary Table 5) suggesting additional physiological consequences upon loss of this synthetic pathway.

Upon deletion of a lipid synthetic pathway, cardiolipin, *E. faecalis* was able to adjust its lipid profile and respond to environmental perturbation. To delve further into the plasticity of the lipidome, we deleted the ability OG1RF to produce L-PG as well as L-PG and CL simultaneously.

### A Strain Deleted for *mprF2* Maintains the Ability to Alter Its Lipid Composition When Supplemented With Exogenous Fatty Acids

**FIGURE 6:**
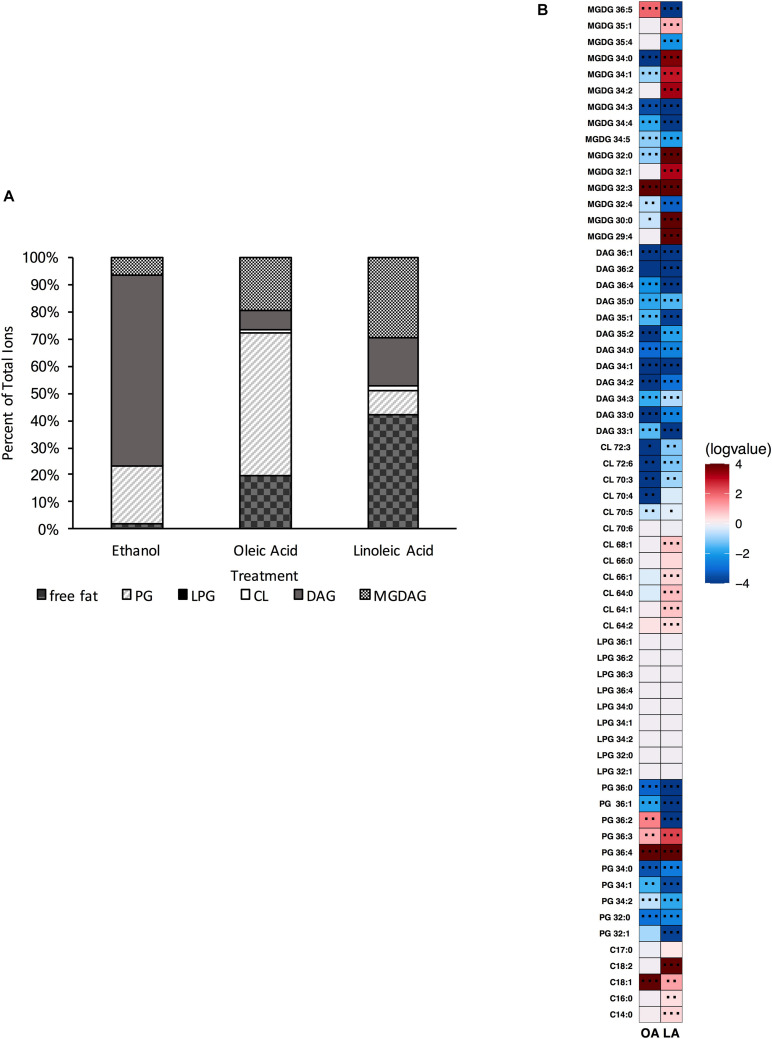
A strain deleted for Δ*mprF2* can alter its lipid composition upon exposure to host fatty acids. **(A)** Percent totals of lipid species in solvent control: free fatty acids (2.1%), PG (20.9%), L-PG (0.0%), CL (0.5%), DAG (69.8%), and MGDG (6.7%), oleic acid: free fatty acids (19.9%), PG (52.5%), L-PG (0.0%), CL 1.0%), DAG (6.9%), and MGDG (19.7%), linoleic acid: free fatty acids (42.2%), PG (8.6%), L-PG (0.0%), CL (2.1%), DAG (17.6%), and MGDG (29.4%). Species were normalized to OD_600 *nm*_. **(B)** Heat-map of fold changes upon fatty acid supplementation vs. solvent control; OA, oleic acid supplementation; LA, linoleic acid supplementation. Shown are the average fold changes of those detected lipid species above limit of detection for *n* = 5 samples. *P = 0.1–0.05; **P = 0.05–0.01; ***P < 0.01.

To examine how *E. faecalis* would respond to loss of L-PG, we deleted *mprF2* (OG1RF_RS03930), which encodes the enzyme responsible for L-PG synthesis. Although *E. faecalis* contains two separate *mprF* genes, a previous study indicated that *mprF2* encoded the major L-PG synthase (Bao et al., 2012). The UHPLC-HRMS analysis confirmed that the Δ*mprF2* strain was unable to produce L-PG, as lipids in this class were below the detection limits under the growth conditions in this study (Supplementary Figure 6 and Figures 6A,B, data not shown). With this loss of L-PG, there was a concomitant significant increase in DAG lipid species and a large decrease in total PG species compared to the parental strain (Supplementary Figure 6 and Figure 6A). There was also a decrease in CL species, similar to how loss of CL resulted in a decrease in L-PG species in Δ*cls1/cls2* (compare Supplementary Figures 3, 6). Importantly, there was no impact on generation time when compared to the parental strain (Supplementary Table 5) despite the inability to produce L-PG.

When provided oleic acid, the Δ*mprF2* strain did not accumulate oleic acid to the same extent as other strains examined in this study (Supplementary Table 4). Levels of the native *cis*-vaccenic acid, C_18:1_*_*cis*_*_11_, remained far more elevated in this strain compared to the others examined (see Discussion). While Δ*mprF2* had increased levels of free C_18:1_ in the membrane when given oleic acid (Figure 6B), we cannot be certain whether this represents an accumulation of native *cis*-vaccenic or oleic acid. Along with these observations, we observed significant increases in PG species (specifically, PG 36:4, PG 36:3, and PG 36:2) and MGDG species (MGDG 32:3 and MGDG 36:5). We note that increases in PG 36:2 and PG 36:3 were also induced in Δ*cls1/cls2 w*hen provided this fatty acid (Figure 4B). Other changes observed in Δ*mprF2* included a decrease in many detected CL and DAG species (Figures 6A,B).

Linoleic acid induced some similar membrane changes, including increases in C_18:1_ and C_18:2_, PG 36:4, PG 36:3 (Figure 6B). However, the total PG composition was reduced upon treatment (Figure 6A). There were also notable decreases in DAG production as seen with oleic acid supplementation. Differing from oleic acid, several CL species including CL 64:2, CL 64:1, CL 64:0, CL 66:1, and CL 68:1 were significantly increased. Furthermore, there was a higher percent of MGDG species in the membrane of the *mprF2* mutant when supplementing with linoleic acid (6.7% control vs. 30% LA Figure 6A), while this trend is not observed in the OG1RF strain (Figure 1A).

Considering the altered membrane of this strain, we were interested in the sensitivity of the Δ*mprF2* strain to membrane disrupting agents compared to the previous cardiolipin synthase mutant.

### Deletion of *mprF2* Impacts Basal Sensitivity to SDS

**FIGURE 7:**
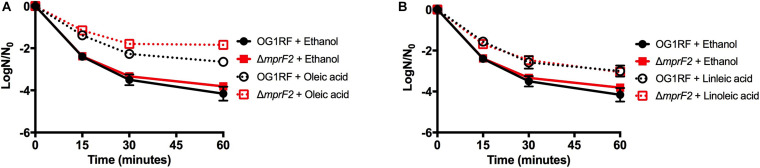
Host fatty acid supplementation protects *mprF2* deficient *E. faecalis* OG1RF from daptomycin challenge. **(A)** All strains supplemented with oleic acid had statistically increased numbers of survivors vs. the solvent control at all-time points analyzed (*P* = 0.002). **(B)** All strains supplemented with linoleic acid had statistically increased numbers of survivors vs. the solvent control at all-time points analyzed (*P* = 0.005). Shown are the averages ± standard deviation for *n* = 3. OG1RF data is re-plotted from Figures 4A,B.

Given that L-PG has been associated with daptomycin resistance and some bacterial species, and that both oleic and linoleic acids have been shown to induce a physiological tolerance to the drug, the relationship between L-PG and daptomycin tolerance upon fatty acid exposure was examined (Hachmann et al., 2009; Bayer et al., 2013; Saito et al., 2014, 2018; Harp et al., 2016; Brewer et al., 2020). Basal sensitivity to high daptomycin concentrations was equivalent in the parental strain and Δ*mprF2* strain (Figures 7A,B). When Δ*mprF2* was grown in media supplemented with oleic or linoleic acid, there was similar increased tolerance to the antibiotic as compared to the parental strain. The role of L-PG in membrane stress responses was then examined by evaluating the sensitivity of the Δ*mprF2* strain to SDS. Surprisingly, the deletion strain was less sensitive to SDS compared to the parental strain (Supplementary Figure 7; see section “Discussion”), implying a role of L-PG in detergent sensitivity.

### OG1RF Lacking *mprF2*, *cls1*, *cls2* Is Viable and Can Alter Its Lipid Content Upon Fatty Acid Supplementation

**FIGURE 8:**
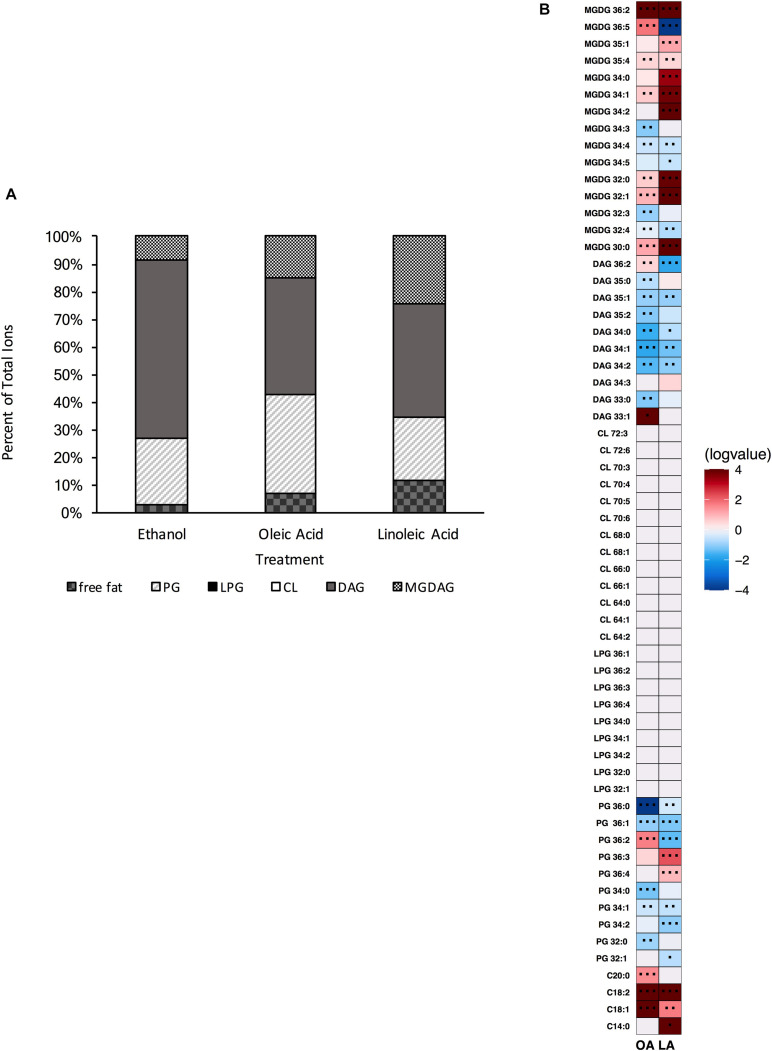
A strain deleted for Δ*mprF2/cls1/cls2* can alter its lipid composition upon exposure to host fatty acids. **(A)** Percent totals of lipid species in solvent control: free fatty acids (2.6%), PG (24.5%), L-PG (0.0%), CL (0.0%), DAG (64.7%), and MGDG (8.3%), oleic acid: free fatty acids (7.0%), PG (35.8%), L-PG (0.0%), CL 0.0%), DAG (64.7%), MGDG (14.8%), linoleic acid: free fatty acids (11.7%), PG (23.0%), L-PG (0.0%), CL (0.0%), DAG (40.8%) MGDG (24.5%). Species were normalized to OD_600 *nm*_. **(B)** Heat-map of fold changes upon fatty acid supplementation vs. solvent control; OA, oleic acid supplementation; LA, linoleic acid supplementation. Shown are the average fold changes of those detected lipid species above limit of detection for *n* = 5 samples. *P = 0.1–0.05; **P = 0.05–0.01; ***P < 0.01.

Thus far, our data have shown that *E. faecalis* can adjust its lipidome in response to genetic disturbances. We wanted to examine further how *E. faecalis* would respond upon loss of two of its three major phospholipid synthetic pathways, L-PG and CL. We therefore generated the triple gene deletion strain Δ*mprF2/cls1/cls2*; this strain grew similar to the original parental strain in laboratory media lacking exogenous fatty acids (Supplementary Table 5). We confirmed the loss of L-PG and CL species via UPLC-HRMS (Supplementary Figure 8 and Figures 8A,B). When lipid species were compared to the original parental strain, there were increases in a variety of DAGs and a single MGDG (Supplementary Figure 7). Indeed DAG species comprised over 64% of the total annotated lipid species examined here, while in the original parental strain they comprised only 22.4% (Figure 1A vs. Figure 8A). There was a large reduction in total PG species detected in Δ*mprF2/cls1/cls2* vs. the original parental strain (23.5 vs. 53.7%, Figure 1A vs. Figure 7A), supportive of significant remodeling of lipid synthetic pathways, despite both CL and L-PG comprising a minor portion of the total lipid profile of the parental strain under control conditions (Figure 1A).

When comparing the basal membrane lipidome to that of cultures supplemented with fatty acids, Δ*mprF2/cls1/cls2* was still capable of altering its lipid composition. When pulsed with oleic acid, there was a significant increase in free fatty acids, in particular C_18:1_, C_18:2_ and C_20:0_, though we note that C_18:2_ was not detected via GC-FAME, likely as levels were below detection limits for this analysis (Supplementary Table 4). Similar to Δ*mprF2*, the accumulation of oleic acid, C_18:1*cis* 9_, as determined via GC-FAME, was far less compared to the other strains examined (Supplementary Table 4). Overall, there was a decrease in the total proportion of the membrane comprised of DAGs and an increase in total proportion of PG species (Figure 8A). The increase in PG species was driven primarily through an increase in PG 36:2 (Figure 8B). There were increases in MGDG species as well, specifically MGDG 36:2, MGDG 36:5, MGDG 32:1, and MGDG 30:0.

Addition of linoleic acid to Δ*mprF2/cls1/cls2* also resulted in an altered profile. Similarly we noted increases in free fatty acids, in particular C_18:2_, which corresponded with the detection of C_18:2*cis*9,12_ via GC-FAME (Supplementary Table 4). MGDGs, as a group, comprised a larger portion of the examined lipid species (approximately 25% vs. control, 8.3% Figure 1A vs. Figure 8A), with specific species like MGDG 34:2, MGDG 36:2, MGDG 32:0, MGDG 34:1 greatly increased (Figure 8B). While PG totals were decreased upon supplementation, PG 36:3 and PG 36:4, which likely contain one and two linoleic tails, respectively, were enriched.

### Loss of Both Lysyl-Phosphatidylglycerol and Cardiolipin Restores Basal Tolerance to Membrane Damaging Agents

As noted above, loss of cardiolipin synthesis resulted in *E. faecalis* being less tolerant to both daptomycin and the general membrane disruptor SDS (Figure 5). However, loss of lysyl-phosphatidylglycerol (Δ*mprF2*) actually increased tolerance to SDS (Supplementary Figure 7). Given these observations, we examined sensitivity Δ*mprF2/cls1/cls2* to these stress agents. Basal daptomycin sensitivity of the triple gene deletion strain was equivalent to the parental strain, unlike Δ*cls1/cls2* (Figures 5A,B); addition of oleic or linoleic acid could induce protection in the triple gene deletion strain. Thus, loss of both L-PG and CL has no impact on daptomycin sensitivity or the induction of protection by eukaryotic fatty acids. Basal sensitivity to the SDS was also similar to the parental strain unlike Δ*cls1/cls2*, although by 60 mins, the survival of Δ*mprF2/cls1/cls2* was reduced (Figure 5C).

## Discussion

Within, we examined the plasticity of the enterococcal lipid composition in response to both environmental perturbations and genetic alterations. We refined a lipidomic approach to identify the most common predicted phospholipids based upon known fatty acid tail and head group content (Supplementary Table 3; Ibbott and Abrams, 1964; Saito et al., 2014, 2018; Hines et al., 2017; Rashid et al., 2017; Tague et al., 2019). We also included analyses of previously reported DAG and MGDG species and fatty acids. We observed that the lipid composition responds quickly upon exposure to the host fatty acids oleic and linoleic acids, resulting in shifts in the proportions of specific PG, CL, L-PG, DAG, and MGDG species. When exposed to exogenous fatty acids, mimicking its environment within humans, OG1RF showed marked increases in lipids containing acyl-chains consisting of the supplemented fatty acid. The parental OG1RF strain responded to exogenous oleic acid by increasing C_18:1_ in the membrane as free fatty acid and as acyl tails onto PG, L-PG, CL, and MGDG. The addition of linoleic acid also increased the amount of free fatty acids dramatically, as well as increasing specific lipid species containing a C_18:2_ acyl-tail. Further, regardless of the presence or absence of either *mprF2*, *cls1* and/or *cls2*, and the resultant differences in their individual lipid species abundance, the basal membrane fatty acid content was unaltered (Supplementary Table 4). This suggests that *E. faecalis* will re-route its phospholipid synthetic machinery such that there are minimal impacts on fatty acid tail composition. This conservation likely aids in maintaining proper membrane fluidity and membrane protein function. These findings are particularly relevant in that *E. faecalis* does not undergo beta-oxidation, therefore the need to uptake these exogenous lipids into the cell for use as a carbon source is void. Future tracing experiments will better determine the fate of these fatty acids as they enter the lipid biosynthesis pathway of this strain.

We do note that there are major differences in membrane lipid composition upon genetic perturbations of lipid synthetic pathways. While L-PG and CL comprised a minor portion of total lipids detected by our methods (Figure 1A), loss of either or both resulted in a basal lipid content being dominated by DAG species and not PG species (Figures 4A, 6A, 8A). Such significant alterations, despite loss of a minor species, demonstrates not only the plasticity of the metabolic capabilities of the organism, but implies the need to maintain proper membrane composition for cellular function. Alterations in membrane composition can have significant impacts on membrane protein function and activity, so this re-wiring of the lipidome likely aids in maintenance of homeostasis (Carson and Daneo-Moore, 1978; Ma and Marquis, 1997; Fozo and Quivey, 2004; Romantsov et al., 2008; Garcia Montes de Oca et al., 2016; Henrich et al., 2016; Lin and Weibel, 2016; Lin et al., 2019). We do note that the genetic perturbations performed are deletion strains, thus we are examining the result of acclimation to genetic manipulation; quick changes in response to gene product depletion will need further investigation.

The increase in DAG species in the various deletion strains compared to the parental, as well as increase in specific DAG species upon fatty acid supplementation, may be supportive of active lipid remodeling. Lipid remodeling in yeast refers to the removal of acyl tails from preformed polar head groups and the addition of new tails (Renne et al., 2015; Patton-Vogt and de Kroon, 2020). In Gram positive bacteria, glycerol phosphate from PG can be transferred to form lipoteichoic acid (in Gram negative bacteria, membrane derived oligosaccharides, MDOs). This generates diacylglycerol, which can be converted to phosphatidic acid and be used to synthesize new lipids (Neuhaus and Baddiley, 2003; Rock, 2008). Thus, large accumulations of DAGs may be indicative of cells actively adjusting their membrane profile (note that cells were actively growing for these analyses). Future metabolic flux and isotope tracing experiments are warranted to better examine lipid turnover.

Deletion of cardiolipin synthase 1 or cardiolipin synthase 2 independently resulted in a strain capable of producing detectable levels of CL. Comparing the parental strain to both single gene deletion strains, there were decreases for all CL species detected (Supplementary Figure 1). However, we do note that both Δ*cls1* and Δ*cls2* had a significant increase in CL species when exposed to oleic acid and linoleic acid, similar to the parental strain (Figures 2B, 3B). This conserved alteration, despite genetic mutation, implies a significant role for CL in acclimating to exogenous host fatty acids in its environment. Further, as increased activity of CL synthase in enterococci and increased levels in liposomes are linked to protection from the antibiotic daptomycin, we noted that deletion of either gene maintained a similar tolerance to the drug as the parental (Arias et al., 2011; Palmer et al., 2011; Mishra et al., 2012; Davlieva et al., 2013; Zhang et al., 2014).

The deletion of both cardiolipin synthase genes resulted in a strain incapable of producing CL (Supplementary Figure 2). The resulting strain produced a membrane vastly different than the parental strain (Figure 4A vs. Figure 1A and Supplementary Figure 3). The Δ*cls1/cls2* strain under basal conditions had a membrane dominated by DAG species, while the parental strain was dominated by PG species. Yet, in the parental strain under the control conditions, CL was only about 1% of the total composition examined. Why then would the composition be so dramatically different upon loss of CL? We note that the deletion strain produces high levels of DAGs, indicative of active lipoteichoic acid synthesis and possible lipid remodeling (Neuhaus and Baddiley, 2003; Rock, 2008; Zhang and Rock, 2008; Theilacker et al., 2009; Theilacker et al., 2011; Van Horn and Sanders, 2012). It could be the loss of CL triggers either wall synthesis and/or lipid recycling, perhaps because protein machinery activity is altered without the lipid present, or lack of CL results in weakening the organism, and wall synthesis is a compensatory response. Further analysis of wall synthesis is needed to confirm this.

Like Δ*cls1/cls2*, Δ*mprF2* also produced a membrane under control conditions vastly different from the parental strain. It had a membrane dominated by DAG species (69.8%) like Δ*cls1/cls2*, whereas the parental strain was dominated by PG species (53.75%; Figures 1A, 4A, 6A). It is important to note that in the parental strain, L-PG comprised only 1.1% of the total composition (Figure 1A). When combining these deletions together, generating Δ*mprF2/cls1/cls2*, again DAG species dominate (Figure 8A). The conserved increase in DAG species suggests the strains alter wall synthesis or phosphatidylglycerol recycling upon loss of phospholipid synthesis (Neuhaus and Baddiley, 2003). Daptomycin has been shown to disrupt wall synthesis (Muller et al., 2016; Grein et al., 2020): if wall synthesis levels were similar in Δ*mprF2/cls1/cls2* and Δ*cls1/cls2*, we would expect these strains to have equivalent sensitivity to the drug. However, that was not what we observed: Δ*cls1/cls2* was much more sensitive to the antibiotic (Figures 5A,B). Further, loss of *mprF2* suppressed Δ*cls1/cls2* sensitivity to the drug (Figures 5A,B). Clearly, further examination of the consequences on other cellular processes, including wall synthesis, upon such perturbation of the lipidome is needed to explain these observations.

While we note that basal fatty acid tail profiles were consistent across our deletion strains, strains lacking *mprF2* did not accumulate oleic acid to the levels observed in the other strains (Supplementary Table 4). Enterococci, like *S. aureus* and *S. pneumoniae*, do not have known “active” import systems for exogenous fatty acids. It is thought that environmental fatty acids interact with the membrane, and passively “flip” in. These fatty acids can then be utilized by the Fak system, resulting in their phosphorylation and use in lipid synthesis, or possibly enter the elongation cycle of fatty acid biosynthesis (Parsons et al., 2014; Cuypers et al., 2019; Gullett et al., 2019). Given this passive mechanism, it is surprising to see such a reduction in oleic acid accumulation specifically: linoleic acid accumulated well in this strain so this is not a generic fatty acid interaction issue (Supplementary Table 4). Even if oleic acid is not used well by the Fak system in a *mprF2* deletion background, we would expect to see it accumulate free in the membrane, and that would be reflected in our GC-FAME analysis. Another possibility is that there is increased oleate hydratase activity in the gene deletion background, which would result in accumulation of C_18:0_, which could then enter fatty acid elongation cycle, generating C_20:0_ (Schroepfer, 1966; Niehaus et al., 1970; Kishino et al., 2013; Radka et al., 2021). We do note an accumulation of C_20:0_ in this strain, but also in other strains, upon supplementation with oleic acid (Supplementary Table 4). Further analyses examining long-term supplementation, and isotope tracing are warranted to better elucidate this observation.

Regardless, all of our mutant strains were able to alter their lipid profiles if provided oleic or linoleic acids. This in turn resulted in induction of tolerance to daptomycin. Previous findings have noted this protection appears independent of membrane fluidity, growth kinetics, or overall membrane charge (Brewer et al., 2020). While daptomycin is known to impact wall synthetic machinery (Muller et al., 2016; Grein et al., 2020), perhaps the addition of oleic or linoleic acid, regardless of genetic background of *E. faecalis*, impacts wall synthesis. We note that all strains examined accumulate free fatty acids within the membrane when given either oleic or linoleic acid: indeed, this seemed to be the only conserved observation across genetic backgrounds (Figures 1B, 2B, 3B, 4B, 6B, 8B). Perhaps the alterations of the lipidome on membrane protein activity is due to free fatty acid accumulation, resulting in stress protection. Ongoing experiments are geared at addressing this.

Loss of L-PG also impacted sensitivity to SDS; however, it rendered Δ*mprF2* more tolerant to the detergent than the parental strain (Supplementary Figure 7). Combining this deletion with Δ*cls1/cls2* actually restored basal tolerance to both SDS and daptomycin to that of a wild type strain. This may be due to alterations in the membrane protein content and/or activity which could in turn be responsible for the improved survival of Δ*mprF2/cls1/cls2* compared to Δ*cls1/cls2* (Figure 5C). Further, differing survival rates upon SDS treatment may be due to altered gene expression in our various deletion strains: a study has shown that SDS can induce a variety of transcriptional changes in enterococci that may aid in acclimation (Solheim et al., 2007). Combined, alterations in the enterococcal lipid content, directly or indirectly, will impact stress responses.

Taken together, we found that supplementation with host fatty acids, oleic acid and linoleic acid, alter the phospholipid profile of *E. faecalis* regardless of genetic perturbation of phospholipid synthetic pathways, supportive of a plastic lipidome and a flexible metabolism. Supplementation with either host fatty acid could induce protection from daptomycin regardless of the presence of *mprF2, cls1*, and *cls2*. Thus, host fatty acid induced daptomycin tolerance during acute exposure to membrane stressors is through a yet to be determined mechanism.

## Data Availability Statement

The datasets generated for this study can be found in Metabolights, study name MTBLS2080: https://www.ebi.ac.uk/metabolights/MTBLS2080.

## Author Contributions

BW performed lipid analyses and subsequent data analyses. JH generated all strains and constructs, prepared cells for fatty acid analyses, performed daptomycin sensitivity assays and growth analyses. BW, EF, WB prepared cells for lipid analyses. ET performed initial lipid analyses. WB performed SDS sensitivity analyses and assisted with figure construction. BW, JH, EF, and SC contributed to the writing of the manuscript. EF and SC directed the project. All authors contributed to the article and approved the submitted version.

## Conflict of Interest

The authors declare that the research was conducted in the absence of any commercial or financial relationships that could be construed as a potential conflict of interest.
