# Supplementary material for: Enterococcus faecalis Readily Adapts Membrane Phospholipid Composition to Environmental and Genetic Perturbation
Source: Front Microbiol. 2021 May 21;12:616045. doi: 10.3389/fmicb.2021.616045 (PMC8177052; doi:10.3389/fmicb.2021.616045)
Supplement: Supplementary file 1 [file Data_Sheet_1.PDF]

Enterococcus faecalis readily adapts membrane phospholipid composition to environmental and genetic perturbation

Brittni M. Woodall[2†], John R. Harp[1†], William T. Brewer[1], Eric D. Tague[2], Shawn R. Campagna[2,3], Elizabeth M. Fozo[1*]

[1]University of Tennessee, Department of Microbiology, Knoxville, TN, USA

[2]University of Tennessee, Department of Chemistry, Knoxville, TN, USA

[2] Biological and Small Molecule Mass Spectrometry Core, University of Tennessee, Knoxville, TN, USA

[†] These authors contributed equally to the work.

*** Correspondence:**
Elizabeth M. Fozo
efozo@utk.edu

Keywords: **lipidome, *mprF2*, lysyl-phosphatidylglycerol, cardiolipin, *cls*, daptomycin**

Supplementary Tables and Figures

Supplementary Table 1. Stains and plasmids used in this study.		
**Strain**	**Relevant genotype or description**	**Source**
Enterococcus faecalis OG1RF	Laboratory strain<br>Rif^R, Fus^R	J. Lemos, University of Florida
Escherichia coli EC1000	Cloning strain for *repA*-dependent plasmids;	G. Dunny, University of Minnesota
E. faecalis CK111/pCF10-101	Conjugative donor strain; harbors non-transferrable pCF10 derivative plasmid	G. Dunny, University of Minnesota<br>Kristich *et al. (*2007) Plasmid 57(2) 131-144
**Plasmid**		
pCJK47	Used for markerless exchange	G. Dunny, University of Minnesota<br>Kristich *et al. (*2007) Plasmid 57(2) 131-144
pJRH1	pCJK47 derivative containing flanking regions of OG1RF_RS01975 (*cls1)*	This work
pJRH2	pCJK47 derivative containing flanking regions of OG1RF_RS06840 (*cls2)*	This work
pMprF2	pCJK47 derivative containing flanking regions of OG1RF_RS03930 (*mprF2*)	This work

Supplementary Table 2. Oligonucleotides used in this study.		
Name	Sequence	Use
**Oligonucleotides used for splicing by overlap extension (SOE) to generate inserts**		
EF1097	GTCCAAGGATTCTCACCATTGATGCAAGGCC	Used to amplify DNA upstream of *cls1*
EF1098	CTGGAGTCATTGTTGTACTATACCATCAATTACGGATTTGGAATATC	Used to amplify DNA upstream of *cls1*
EF1099	CGTAATTGATGGTATACGTACAACAATGACTCCAGAAGTTGTTCGTGAC	Used to amplify DNA downstream of *cls1*
EF1105	GACTGTCCATGGTATGTTGCACAGCTTCCATCG	Used to amplify DNA downstream of *cls1*
EF1103	CAAGACTGGTGACCAACGTCATCATCCATGCTAAAACGCTGG	Used to amplify DNA upstream of *cls2*
EF1108	CCCAAGCCCGGGCGATTGACCAGGACCACTTAAAACTCC	Used to amplify DNA upstream of *cls2*
EF1113	GGTCACGGATTCTCCAAACAAGGTAACC	Used to amplify DNA downstream of *cls2*
EF1102	CATGGATGATGACGTTGGTCACCAGTCTTGTAATCAATAAATCAGCAGTG	Used to amplify DNA downstream of *cls2*
**Oligonucleotides used for Gibson assembly**		
EF1449	CAATCACTAGTGAATTCGCGGCCGCCACGGCGATATCGG	Generate overhangs on pCJK47 for *cls1*
EF1450	CAATCGAATTCCCGCGGCCGCTCTAGAACTAGCGATTCTGAAATCAC	Generate overhangs on pCJK47 for *cls1*
EF1451	CAGAATCGCTAGTTCTAGAGCGGCCGCGGGAATTCGATTGT	Generate overhangs on *cls1* insert
EF1452	CATATGGATCCGATATCGCCGTGGCGGCCGCGAATTCACTAGTGATTGACTG	Generate overhangs on *cls1* insert
EF1453	GGGAATCACTAGTGAATTCGCGGCCGCACGGCGATATCGGATCC	Generate overhangs on pCJK47 for *cls2*
EF1454	CCGTGACCAATCGAATTCCCGCGGCCGCTCTAGAACTAGCGATTCTGAAATCAC	Generate overhangs on pCJK47 for *cls2*
EF1455	CAGAATCGCTAGTTCTAGAGCGGCCGCGGGAATTCGATTGG	Generate overhangs on *cls2* insert
EF1456	CATATGGATCCGATATCGCCGTGCGGCCGCGAATTCACTAGTGATTCCCAAG	Generate overhangs on *cls2* insert
EF1601	GACAAAACCCCTAATAATTCTTTTGCTTCATCCATGCCCGGGTACCATGGCATGCTAAGCTTGATTTTCGTTC	Generate overhangs on pCJK47 for *mprF2*
EF1602	CTTTTGATCAAAAACAGGATTTTCTCTAGAACTAGCGATTCTGAAATCACCATTTAAAAAACTC	Generate overhangs on pCJK47 for *mprF2*
EF1603	GAGTTTTTTAAATGGTGATTTCAGAATCGCTAGTTCTAGAGAAAATCCTGTTTTTGATCAAAAG	Generate overhangs on DNA upstream of *mprF2*
EF1604	CAATCCAGCTACTTTTAGAATAAAGTGTATAGCAACAACAATAATTGAGACCGCAATAACAAAC	Generate overhangs on DNA upstream of *mprF2*
EF1605	GTTTGTTATTGCGGTCTCAATTATTGTTGTTGCTATACACTTTATTCTAAAAGTAGCTGGATTG	Generate overhangs on DNA downstream of *mprF2*
EF1606	GAACGAAAATCAAGCTTAGCATGCCATGGTACCCGGGCATGGATGAAGCAAAAGAATTATTAGGGGTTTTGTC	Generate overhangs on DNA downstream of *mprF2*

Supplementary Table 3. Lipid Compound List for UPLC-HRMS Identification with Tail Composition		
Polarity	**Lipid**	**Formula**
-	CL 64:2	C73H138O17P2
-	CL 64:0	C73H142O17P2
-	CL 64:1	C73H140O17P2
-	CL 66:0	C75H146O17P2
-	CL 66:1	C75H144O17P2
-	CL 68:0	C77H150O17P2
-	CL 68:1	C77H148O17P2
-	CL 70:0	C79H154O17P2
-	CL 70:1	C79H152O17P2
-	CL 70:3	C79H148O17P2
-	CL 70:4	C79H146O17P2
-	CL 70:5	C79H144O17P2
-	CL 70:6	C79H142O17P2
-	CL 70:7	C79H140O17P2
-	CL 72:5	C81H148O17P2
-	CL 72:6	C81H146O17P2
-	CL 56:0	C65H126O17P2
-	CL 72:8	C81H142O17P2
-	CL 72:7	C81H144O17P2
-	CL 72:4	C81H150O17P2
-	CL 72:3	C81H152O17P2
-	CL 72:2	C81H154O17P2
-	CL 72:1	C81H156O17P2
-	CL 72:0	C81H158O17P2
-	LPG 32:1	C44H85N2O11P
-	LPG 34:0	C46H91N2O11P
-	LPG 34:1	C46H89N2O11P
-	LPG 34:2	C46H87N2O11P
-	LPG 36:0	C48H95N2O11P

-	LPG 36:1	C48H93N2O11P
-	LPG 36:3	C48H89N2O11P
-	LPG 36:4	C48H87N2O11P
-	LPG 32:0	C44H87N2O11P
-	LPG 36:2	C48H91N2O11P
-	PG 32:0	C38H75O10P
-	PG 32:1	C38H73O10P
-	PG 34:0	C40H79O10P
-	PG 34:1	C40H77O10P
-	PG 34:2	C40H75O10P
-	PG 36:3	C42H77O10P
-	PG 36:4	C42H75O10P
-	PG 36:2	C42H79O10P
-	PG 36:1	C42H81O10P
-	PG 36:0	C42H83O10P
-	PG 16:0	C22H43O10P
+	DAG 36:2	C39H75NO5
+	DAG 36:4	C39H71NO5
+	DAG 36:0	C39H79NO5
+	DAG 36:1	C39H77NO5
+	DAG 35:0	C38H77NO5
+	DAG 35:1	C38H75NO5
+	DAG 35:2	C38H73NO5
+	DAG 34:0	C37H75NO5
+	DAG 34:1	C37H73NO5
+	DAG 34:2	C37H71NO5
+	DAG 33:0	C36H73NO5
+	DAG 33:1	C36H71NO5
+	DAG 33:2	C36H69NO5
+	DAG 35:3	C38H71NO5
+	DAG 34:3	C37H69NO5
+	DAG 33:3	C36H67NO5
+	DAG 35:4	C38H69NO5
+	DAG 34:4	C37H67NO5

+	MGDG 28:0	C37H73NO10
+	MGDG 28:1	C37H68O10NH3
+	MGDG 28:2	C37H66O10NH3
+	MGDG 28:3	C37H64O10NH3
+	MGDG 28:4	C37H62O10NH3
+	MGDG 28:5	C37H60O10NH3
+	MGDG 28:6	C37H58O10NH3
+	MGDG 29:0	C38H72O10NH3
+	MGDG 29:1	C38H70O10NH3
+	MGDG 29:2	C38H68O10NH3
+	MGDG 29:3	C38H66O10NH3
+	MGDG 29:4	C38H64O10NH3
+	MGDG 29:5	C38H62O10NH3
+	MGDG 29:6	C38H60O10NH3
+	MGDG 30:0	C39H74O10NH3
+	MGDG 30:1	C39H72O10NH3
+	MGDG 30:2	C39H70O10NH3
+	MGDG 30:3	C39H68O10NH3
+	MGDG 30:4	C39H66O10NH3
+	MGDG 30:5	C39H64O10NH3
+	MGDG 30:6	C39H62O10NH3
+	MGDG 31:0	C40H76O10NH3
+	MGDG 31:1	C40H74O10NH3
+	MGDG 31:2	C40H72O10NH3
+	MGDG 31:3	C40H70O10NH3
+	MGDG 31:4	C40H68O10NH3
+	MGDG 31:5	C40H66O10NH3
+	MGDG 31:6	C40H64O10NH3
+	MGDG 32:0	C41H78O10NH3
+	MGDG 32:1	C41H76O10NH3
+	MGDG 32:2	C41H74O10NH3
+	MGDG 32:3	C41H72O10NH3
+	MGDG 32:4	C41H70O10NH3
+	MGDG 32:5	C41H68O10NH3

+	MGDG 32:6	C41H66O10NH3
+	MGDG 33:0	C42H80O10NH3
+	MGDG 33:1	C42H78O10NH3
+	MGDG 33:2	C42H76O10NH3
+	MGDG 33:3	C42H74O10NH3
+	MGDG 33:4	C42H72O10NH3
+	MGDG 33:5	C42H70O10NH3
+	MGDG 33:6	C42H68O10NH3
+	MGDG 34:0	C43H82O10NH3
+	MGDG 34:1	C43H80O10NH3
+	MGDG 34:2	C43H78O10NH3
+	MGDG 34:3	C43H76O10NH3
+	MGDG 34:4	C43H74O10NH3
+	MGDG 34:5	C43H72O10NH3
+	MGDG 34:6	C43H70O10NH3
+	MGDG 35:0	C44H84O10NH3
+	MGDG 35:1	C44H82O10NH3
+	MGDG 35:2	C44H80O10NH3
+	MGDG 35:3	C44H78O10NH3
+	MGDG 35:4	C44H76O10NH3
+	MGDG 35:5	C44H74O10NH3
+	MGDG 35:6	C44H72O10NH3
+	MGDG 36:0	C45H86O10NH3
+	MGDG 36:1	C45H84O10NH3
+	MGDG 36:2	C45H82O10NH3
+	MGDG 36:3	C45H80O10NH3
+	MGDG 36:4	C45H78O10NH3
+	MGDG 36:5	C45H76O10NH3
+	MGDG 36:6	C45H74O10NH3
+	MGDG 37:0	C46H88O10NH3
+	MGDG 37:1	C46H86O10NH3
+	MGDG 37:2	C46H84O10NH3
+	MGDG 37:3	C46H82O10NH3
+	MGDG 37:4	C46H80O10NH3

+	MGDG 37:5	C46H78O10NH3
+	MGDG 37:6	C46H76O10NH3
+	MGDG 38:0	C47H88O10NH3
+	MGDG 38:1	C47H86O10NH3
+	MGDG 38:2	C47H84O10NH3
+	MGDG 38:3	C47H82O10NH3
+	MGDG 38:4	C47H80O10NH3
+	MGDG 38:5	C47H78O10NH3
+	MGDG 38:6	C47H76O10NH3
+	MGDG 40:0	C49H92O10NH3
+	MGDG 40:1	C49H90O10NH3
+	MGDG 40:2	C49H88O10NH3
+	MGDG 40:3	C49H86O10NH3
+	MGDG 40:4	C49H84O10NH3
+	MGDG 40:5	C49H82O10NH3
+	MGDG 40:6	C49H80O10NH3
+	MGDG 42:0	C51H96O10NH3
+	MGDG 42:1	C51H94O10NH3
+	MGDG 42:2	C51H92O10NH3
+	MGDG 42:3	C51H90O10NH3
+	MGDG 42:4	C51H88O10NH3
+	MGDG 42:5	C51H86O10NH3
+	MGDG 42:6	C51H84O10NH3
+	MGDG 44:0	C53H100O10NH3
+	MGDG 44:1	C53H98O10NH3
+	MGDG 44:2	C53H96O10NH3
+	MGDG 44:3	C53H94O10NH3
+	MGDG 44:4	C53H92O10NH3
+	MGDG 44:5	C53H90O10NH3
+	MGDG 44:6	C53H88O10NH3
-	C14:0	C14H28O2
-	C12:0	C12H24O2
-	C16:0	C16H32O2
-	C16:1	C16H30O2

-	C18:0	C18H36O2
-	C18:1	C18H34O2
-	C18:2	C18H32O2
-	C20:0	C20H40O2
-	C20:4	C20H32O2
-	C20:5	C20H30O2
-	C22:6	C22H32O2
-	C20:1	C20H38O2

Supplementary Table 4. *E. faecalis* OG1RF, Δ*mprF2,* Δ*cls1*, Δ*cls2*, Δ*cls1*/*cls2*, and Δ*mprF2*/*cls1*/*cls2* membrane fatty acid composition during exponential phase growth via GC-FAME (Microbial ID, Inc.)

	% of total membrane content (Avg. ± SD)																	
	Ethanol						Oleic acid - $C_{18:1\ cis\ 9}$						Linoleic acid- C18:2 $_{cis\ 9,12}$					
Fatty acid	OG1RF	Δ*mprF2*	Δ*cls1*	Δ*cls2*	Δ*cls1*/ Δ*cls2*	Δ*mprF2/cls1/cls2*	OG1RF	Δ*mprF2*	Δ*cls1*	Δ*cls2*	Δ*cls1 /cls2*	Δ*mprF2/ cls1/cls2*	OG1RF	Δ*mprF2*	Δ*cls1*	Δ*cls2*	Δ*cls1 /cls2*	Δ*mprF2/ cls1/cls2*
$C_{12:0}$	1.8 ± 0.3	1.2 ± 0.1	1.6 ± 0.1	1.7 ± 0.4	1.7 ± 0.2	1.3 ± 0.3	0.7 ± 0.1	1.2 ± 0.1	0.7 ± 0.1	0.8 ± 0.1	0.6 ± 0.1	1.6 ± 0.2	1.2 ± 0.1	1.4 ± 0.2	1.0 ± 0.1	1.1 ± 0.04	1.0 ± 0.1	1.3 ± 0.2
$C_{14:0}$	4.8 ± 0.1	4.6 ± 0.2	4.6 ± 0.04	4.4 ± 0.4	4.2 ± 0.1	4.4 ± 0.4	2.5 ± 0.2	4.2 ± 0.1	2.4 ± 0.1	2.4 ± 0.02	1.6 ± 0.1	4.0 ± 0.2	3.7 ± 0.1	4.3 ± 0.4	3.6 ± 0.1	3.5 ± 0.1	3.0 ± 0.1	3.5 ± 0.3
$C_{16:1\ cis\ 9}$	7.2 ± 0.1	6.6 ± 0.3	6.7 ± 0.1	7.1 ± 0.4	6.5 ± 0.2	7.0 ± 0.6	3.8 ± 0.3	5.7 ± 0.3	3.8 ± 0.1	4.0 ± 0.1	2.7 ± 0.2	5.7 ± 0.4	6.1 ± 0.04	5.9 ± 0.2	5.9 ± 0.2	6.1 ± 0.1	5.4 ± 0.1	5.3 ± 0.3
$C_{16:0}$	37.8 ± 0.4	38.6 ± 0.4	37.5 ± 0.3	36.0 ± 0.2	37.1 ± 0.3	37.3 ± 0.8	19.5 ± 0.6	31.1 ± 0.1	18.6 ± 0.5	18.0 ± 0.3	14.7 ± 0.6	29.5 ± 0.1	29.6 ± 0.5	31.0 ± 2.1	29.2 ± 0.2	29.0 ± 0.6	26.8 ± 0.4	29.0 ± 1.1
$C_{18:1\ cis\ 9}$	1.7 ± 0.2	ND	1.3 ± 0.1	1.4 ± 0.1	1.3 ± 0.1	0.3 ± 0.5	46.7 ± 1.7	11.1 ± 1.0	47.1 ± 1.7	42.2 ± 6.0	59.8 ± 2.1	11.5 ± 4.3	ND	ND	ND	ND	ND	ND
$C_{18:1\ cis\ 11}$	38.3 ± 0.5	39.6 ± 0.1	39.2 ± 0.4	41.3 ± 0.9	40.2 ± 0.6	41.9 ± 0.9	19.0 ± 0.6	29.9 ± 0.3	18.3 ± 0.4	19.0 ± 0.7	14.8 ± 0.4	28.0 ± 0.4	28.6 ± 0.5	26.6 ± 1.7	28.4 ± 0.2	30.8 ± 0.9	29.2 ± 0.8	27.0 ± 0.8
$C_{18:0}$	5.0 ± 0.1	5.5 ± 0.2	5.5 ± 0.1	4.6 ± 0.1	5.4 ± 0.2	4.9 ± 0.3	2.9 ± 0.2	3.8 ± 0.2	2.8 ± 0.3	2.6 ± 0.1	2.5 ± 0.1	3.6 ± 0.2	4.3 ± 0.1	4.2 ± 0.5	4.4 ± 0.3	4.0 ± 0.1	4.0 ± 0.2	4.1 ± 0.1
$C_{18:2\ cis\ 9,12}$	ND	ND	ND	ND	ND	ND	ND	ND	ND	ND	ND	ND	24.0 ± 0.9	23.4 ± 3.1	28.4 ± 0.2	22.0 ± 1.4	27.8 ± 0.9	26.1 ± 2.7
$C_{17:0\ 2OH}$	2.0 ± 0.2	2.8 ± 0.4	2.1 ± 0.3	1.9 ± 0.3	1.9 ± 0.4	1.6 ± 1.4	1.1 ± 0.1	1.8 ± 0.3	1.0 ± 0.2	1.1 ± 0.3	0.7 ± 0.1	1.3 ± 0.2	1.6 ± 0.1	1.9 ± 0.2	1.8 ± 0.03	1.7 ± 0.1	1.8 ± 0.1	2.2 ± 0.2
$C_{19:0\ cyclo\ 11}$	ND	0.8 ± 0.0	ND	ND	ND	1.0 ± 0.2	ND	0.8 ± 0.1	ND	ND	ND	1.3 ± 0.1	ND	0.7 ± 0.1	ND	ND	ND	1.2 ± 0.1
$C_{20:0}$	ND	ND	ND	ND	ND	ND	3.2 ± 0.3	10.0 ± 1.0	4.8 ± 1.2	9.3 ± 5.1	1.8 ± 1.6	13.0 ± 3.3	ND	ND	ND	ND	ND	ND
Others[e]	1.3 ± 0.6	0.4 ± 0.7	1.5 ± 0.1	1.5 ± 0.6	1.6 ± 0.3	0.3 ± 0.6	0.6 ± 0.2	0.5 ± 0.5	0.6 ± 0.1	0.6 ± 0.2	0.8 ± 0.1	0.5 ± 0.4	0.9 ± 0.3	0.7 ± 1.0	0.7 ± 0.2	1.7 ± 0.8	1.1 ± 0.2	0.5 ± 0.4
Sat/Unsat	1.1 ± 0.02	1.2 ± 0.04	1.1 ± 0.01	1.0 ± 0.03	1.1 ± 0.01	1.0 ± 0.02	0.4 ± 0.5	1.1 ± 0.1	0.4 ± 1.0	0.5 ± 0.9	0.3 ± 0.9	1.2 ± 0.2	0.7 ± 0.5	0.8 ± 0.6	0.7 ± 0.7	0.7 ± 0.4	0.6 ± 0.5	0.7 ± 0.5
C_{10} - C_{17} / C_{18} - C_{20}[f]	1.2 ± 0.01	1.2 ± 0.01	1.1 ± 0.01	1.1 ± 0.05	1.1 ± 0.02	1.1 ± 0.06	0.4 ± 0.4	0.8 ± 0.02	0.4 ± 0.3	0.4 ± 0.1	0.3 ± 0.2	0.74 ± 0.03	0.7 ± 0.5	0.8 ± 0.6	0.7 ± 0.5	0.7 ± 0.3	0.6 ± 0.4	0.7 ± 0.6

[a]Shown are averages ± standard deviations from three independent cultures. ND=not detected.
[b]Ethanol was added at a final concentration of 0.1%.
[c]Oleic acid was added at a final concentration of 20 μg ml^{-1}.
[d]Linoleic acid was added at a final concentration of 10 μg ml^{-1}.
[e]Others indicates fatty acids that comprised <1% of the total membrane content.
[f]Fatty acid length ratio includes both saturated and unsaturated fatty acid.

Supplementary Table 5. Exponential phase generation times if given long term			
	Generation times in medium constituent (min)[a]		
Strain	Ethanol[b]	Oleic acid[c]	Linoleic acid[d]
OG1RF	37.0 ± 0.3	47.0 ± 1.1	88.2 ± 8.3
Δ*mprF2*	38.3 ± 1.0	36.2 ± 1.5	74.8 ± 6.9
Δ*cls1*	36.7 ± 1.5	47.2 ± 0.2	77.5 ± 1.1
Δ*cls2*	35.9 ± 1.0	50.1 ± 2.3	86.0. ± 6.8
Δ*cls1*/*cls2*	35.0 ± 0.9	52.2 ± 0.4	252.9 ± 98.5
Δ*mprF2*/*cls1*/*cls2*	38.8 ± 5.1	39.7 ± 3.5	195.5 ± 22.2

[a]Shown are averages ± standard deviations from three independent cultures.

[b]Ethanol was added at a final concentration of 0.1%.

[c]Oleic acid was added at a final concentration of 20 μg ml^{-1}.

[d]Linoleic acid was added at a final concentration of 10 μg ml^{-1}.

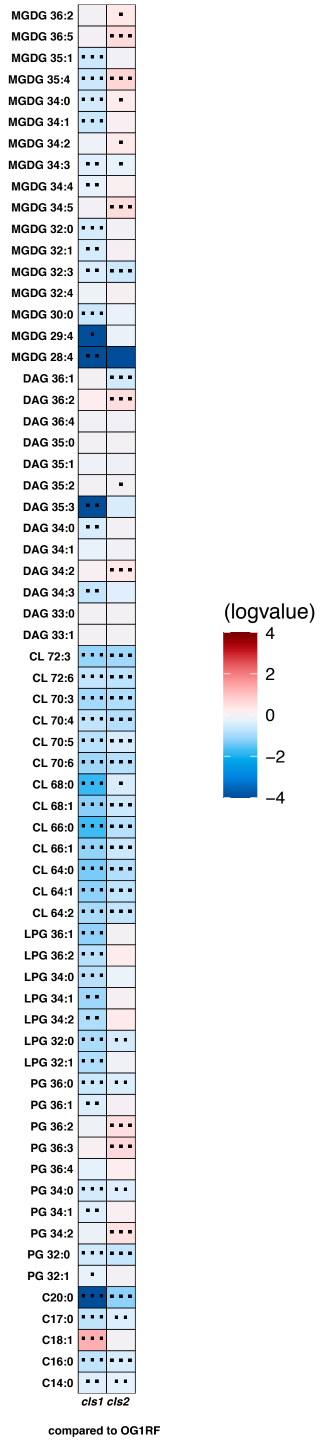

Supplementary Figure 1. Heat-map of *Δcls1* and *Δcls2* compared to OG1RF in solvent control. Shown are the average fold changes of those detected lipid species above limit of detection for *n*=5. * *P* = 0.1-0.05; ** *P* = 0.05-0.01; *** *P*<0.01.

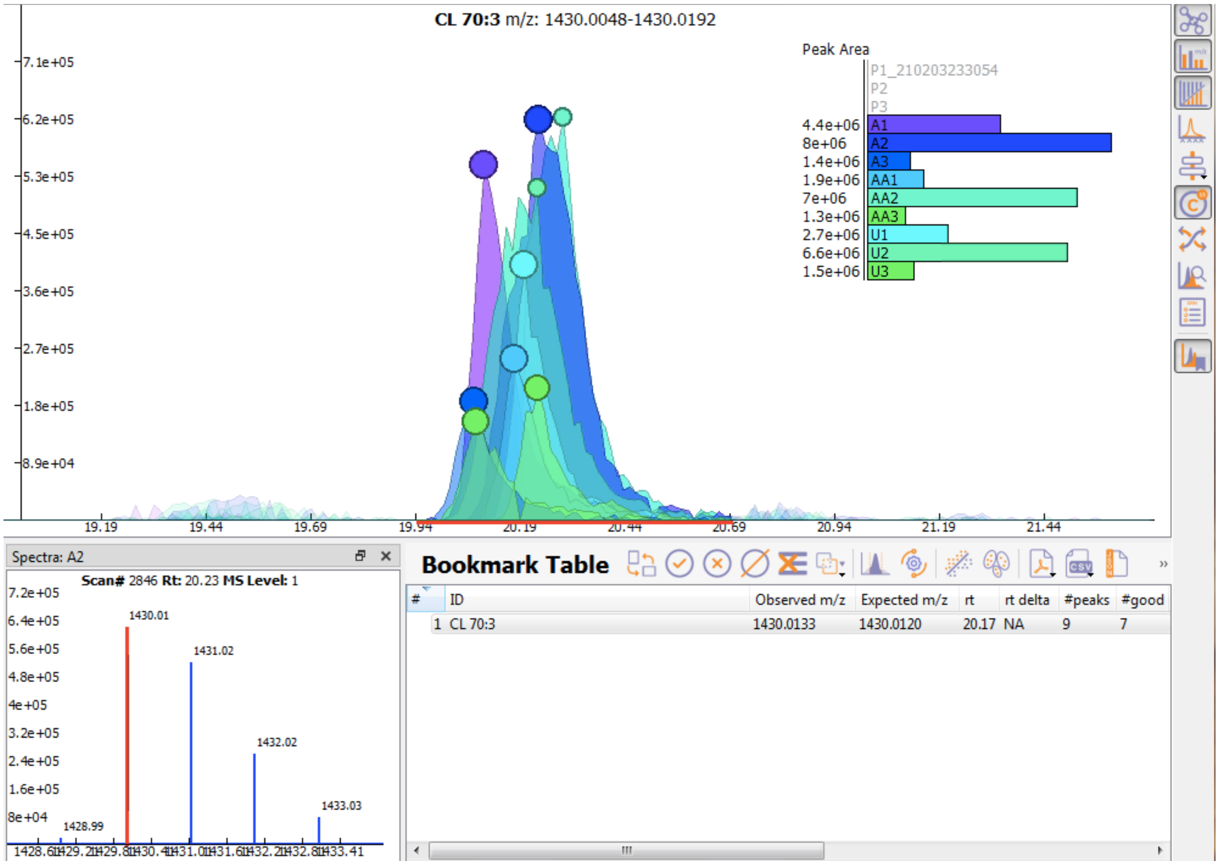

#	ID	Observed m/z	Expected m/z	rt	rt delta	#peaks	#good
1	CL 70:3	1430.0133	1430.0120	20.17	NA	9	7

Supplementary Figure 2. Chromatogram of OG1RF, *Δcls1*, Δcls2, and *Δcls1/cls2* displaying cardiolipin 70:3. Sample ID: A1: OG1RF ETOH; A2: OG1RF OA; A3: OG1RF LA; AA1: *Δcls2* ETOH; AA2: Δcls2 OA; AA3: *Δcls2* LA; U1: *Δcls1* ETOH; U2: *Δcls1* OA, U3: *Δcls1* LA; P1: *Δcls1/cls2*: ETOH; P2: *Δcls1/cls2* OA; P3: *Δcls1/cls2* LA. ETOH: solvent control; OA: oleic acid exposure; LA: linoleic acid exposure. See Materials and Methods for details.

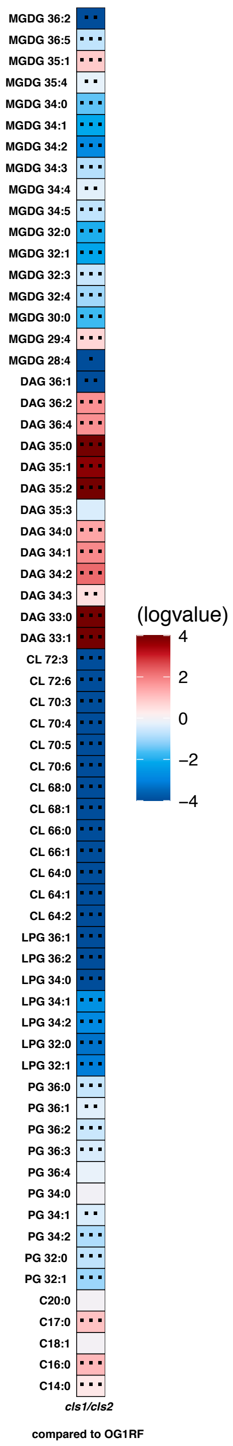

Supplementary Figure 3. Heat-map of *Δcls1/Δcls 2* compared to OG1RF in solvent control. Shown are the average fold changes of those detected lipid species above limit of detection for *n*=5. * *P* = 0.1-0.05; ** *P* = 0.05-0.01; *** *P*<0.01.

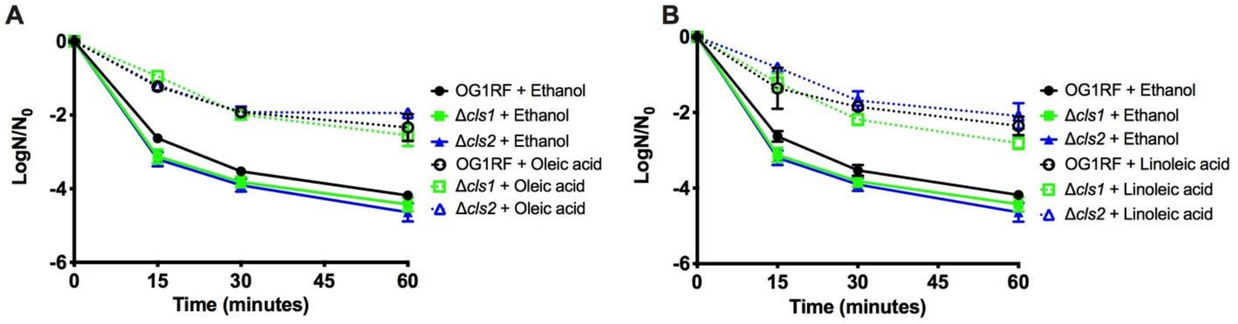

Supplementary Figure 4. Short-term fatty acid supplementation with host fatty acids protects OG1RF *Δcls1* and *Δcls2* from daptomcyin challenge. Supplementation with oleic acid (A) or linoleic acid (B) for either *Δcls1* or *Δcls2* had statistically increased numbers of survivors versus the solvent control at all time points assessed ($P = 0.0001$). Shown are the average ± standard deviation for $n = 3$.

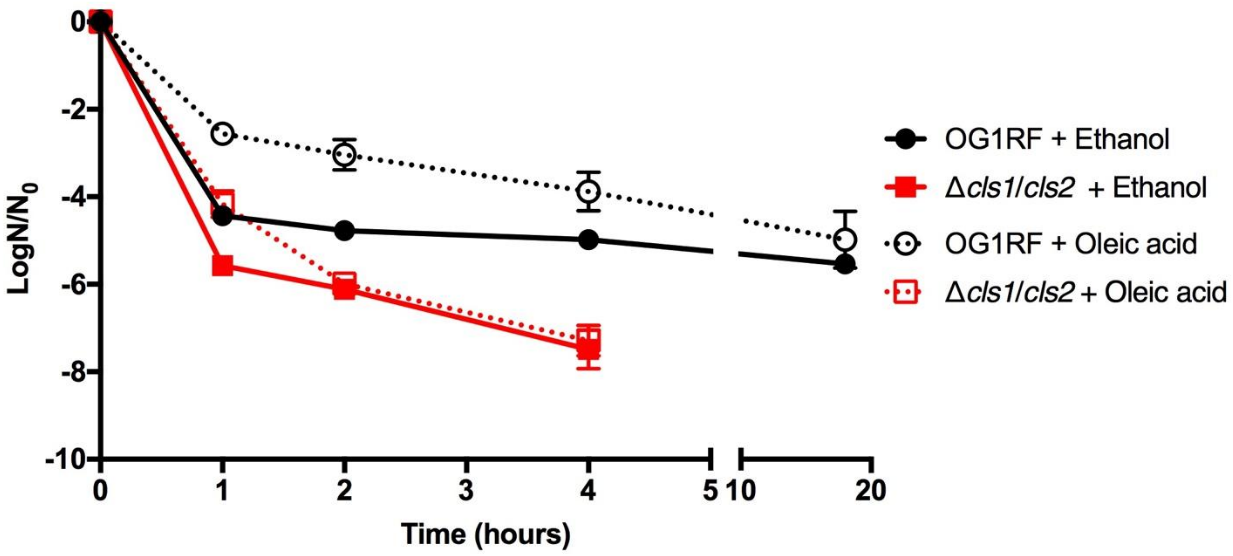

Supplementary Figure 5. Short-term supplementation with oleic acid does not protect *Δcls1/cls2* from extended daptomycin challenge. The *Δcls1/cls2* had no viable colonies after 4 h of exposure. Shown are the averages ± standard deviation for *n* = 3.

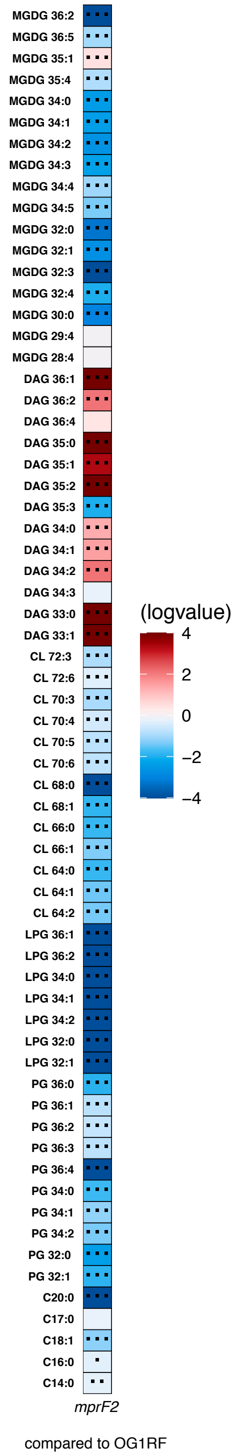

Supplementary Figure 6. Heat-map of *ΔmprF2* compared to OG1RF in solvent control. Shown are the average fold changes of those detected lipid species above limit of detection for *n* = 5. * *P* = 0.1-0.05; ** *P* = 0.05-0.01; *** *P*<0.01.

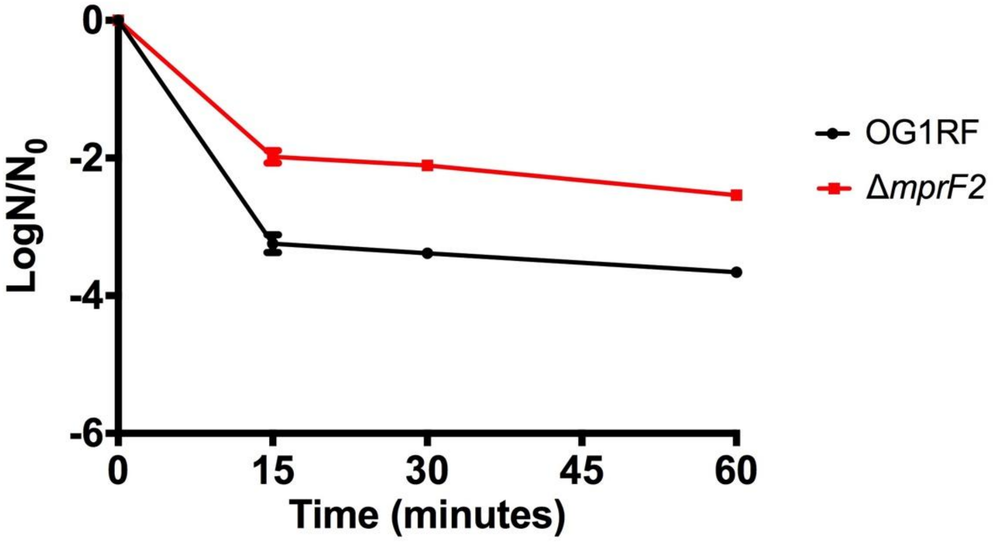

Supplementary Figure 7. *ΔmprF2* survives SDS challenge better than the parental strain. Experimental details are found within the Materials and Methods. *ΔmprF2* had statistically more survivors (*P*<0.01) than the parental strain at each time point. Shown are the averages ± standard deviation for *n* = 3.

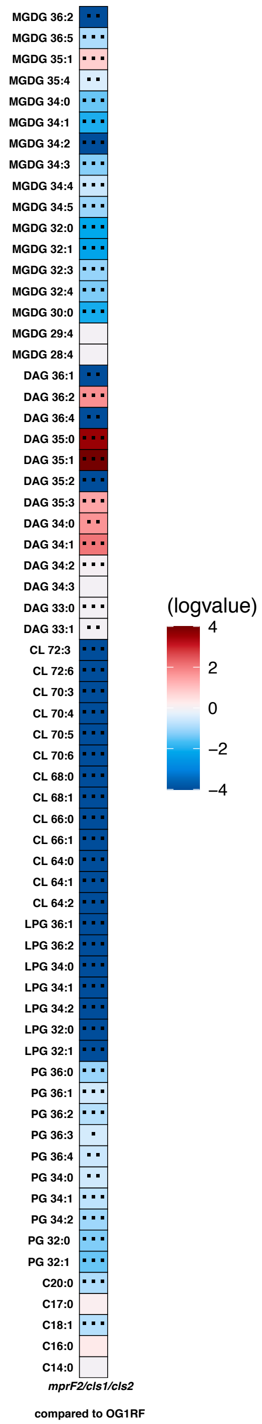

Supplementary Figure 8. Heat-map of *ΔmprF2/cls1/cls2* compared to OG1RF in solvent control. Shown are the average fold changes of those detected lipid species above limit of detection for *n* = 5. * *P* = 0.1-0.05; ** *P* = 0.05-0.01; *** *P*<0.01.
